# Water use efficiency responses to fluctuating soil water availability in contrasting commercial sugar beet varieties

**DOI:** 10.3389/fpls.2023.1119321

**Published:** 2023-03-09

**Authors:** Georgina E. Barratt, Erik H. Murchie, Debbie L. Sparkes

**Affiliations:** Division of Plant and Crop Sciences, School of Biosciences, University of Nottingham, Nottingham, United Kingdom

**Keywords:** sugar beet, water use, drought, stomata, carbon isotopes, crop, yield, agriculture

## Abstract

Many areas of sugar beet production will face hotter and drier summers as the climate changes. There has been much research on drought tolerance in sugar beet but water use efficiency (WUE) has been less of a focus. An experiment was undertaken to examine how fluctuating soil water deficits effect WUE from the leaf to the crop level and identify if sugar beet acclimates to water deficits to increase WUE in the longer term. Two commercial sugar beet varieties with contrasting upright and prostrate canopies were examined to identify if WUE differs due to contrasting canopy architecture. The sugar beet were grown under four different irrigation regimes (fully irrigated, single drought, double drought and continually water limited) in large 610 L soil boxes in an open ended polytunnel. Measurements of leaf gas exchange, chlorophyll fluorescence and relative water content (RWC) were regularly undertaken and stomatal density, sugar and biomass yields and the associated WUE, SLW and Δ^13^C were assessed. The results showed that water deficits generally increase intrinsic (WUE_i_) and dry matter (WUE_DM_) water use efficiency but reduce yield. Sugar beet recovered fully after severe water deficits, as assessed by leaf gas exchange and chlorophyll fluorescence parameters and, except for reducing canopy size, showed no other acclimation to drought, and therefore no changes in WUE or drought avoidance. Spot measurements of WUE_i,_ showed no differences between the two varieties but the prostrate variety showed lower Δ^13^C values, and traits associated with more water conservative phenotypes of a lower stomatal density and greater leaf RWC. Leaf chlorophyll content was affected by water deficit but the relationship with WUE was unclear. The difference in Δ^13^C values between the two varieties suggests traits associated with greater WUE_i_ may be linked to canopy architecture.

## 1 Introduction

Climate change is causing hotter and drier summers in many areas of Europe (Evans, 2017) with crop yields increasingly limited by water availability (Angert et al., 2005). A sufficient supply of water is crucial to maximizing plant yield because dry matter (DM) accumulation is directly proportional to water use in most environments. This is because solar radiation drives both photosynthesis and transpiration (Tanner and Sinclair, 1983). The relationship between photosynthesis and transpiration, known as water use efficiency (WUE) can be assessed at a range of scales. At the leaf level by assessing carbon uptake in relation to stomatal conductance (g_s_), known as intrinsic water use efficiency (WUE_i_) (Farquhar et al., 1989) and the crop level by calculating the ratio of DM accumulated to water used by the crop (WUE_DM_) (Boyer, 1996). In crops, WUE can be further defined to consider only the DM of the harvested product (Fageria et al., 2006). Increasing WUE can be achieved through manipulation of three key processes which operate from the leaf to the crop level, (i) reducing water loss, for example through soil evaporation and water passing beyond the root zone, (ii) reducing the rate of transpiration to carbon fixation and (iii) increasing the harvest index (Condon et al., 2002). A large number of traits are associated with these processes and those that influence photosynthesis and stomatal anatomy are of particular interest (Leakey et al., 2019). Environmental factors are also key to WUE with light, temperature and soil water availability affecting plant water use and carbon fixation (Hatfield and Dold, 2019). The relationship between carbon fixation and water use means that increasing crop WUE can be a trade-off between photosynthesis and transpiration (Blum, 2005). Despite this trade-off there has been success in breeding commercial wheat varieties which are more efficient in their use of water, without reducing yield potential under optimal conditions (Condon et al., 2002; Condon et al., 2004). This was achieved through identifying differences in carbon isotope discrimination (Δ^13^C) which is inversely related to WUE_i_ (Farquhar and Richards, 1984) and allowed breeding for specific environments where water conservation or fast growth was required. As breeders move away from focusing solely on maximizing yield and look for traits which increase crop resilience, including those associated with WUE, it is important to understand if there are differences in such traits in sugar beet. Although there has been extensive work on drought tolerance in sugar beet (Ober and Luterbacher, 2002; Ober et al., 2004; Ober et al., 2005) WUE_i_ was not assessed and may be a useful trait when developing more WUE varieties for the regions in which climate change is leading to reduced water availability.

Sugar beet varieties are developed for a range of markets around the world, from the dry climates of the Middle East to the temperate climates of Europe and North America (Morillo-Velarde and Ober, 2008). Although there are many studies looking at the effects of irrigation on sugar beet in drier climates (Mohammadian et al., 2005; Hassanli et al., 2010; Topak et al., 2011; Li et al., 2019), in much of Europe, irrigation is not economically feasible (Rezbova et al., 2013) and sugar beet WUE must be increased to maximize the use of rainfall to reach the crop’s full yield potential (Hoffmann and Kenter, 2018). Despite a maritime ancestry, which makes sugar beet more drought tolerant than many major crop species (Dunham, 1993), yield losses are still evident under drought with unirrigated losses in Europe ranging from 15-40% depending on the regional climate and soil type (Pidgeon et al., 2001). Significant variations in drought tolerance have been identified within the sugar beet germplasm and Beta gene bank accessions driving further work to understand the level of drought tolerance in breeders’ lines (Ober and Luterbacher, 2002). The work of Ober et al. (2004) compared sugar beet breeding lines and varieties grown for a range of climates to look at the drought tolerance index (DTI) (the fraction of irrigated yield maintained under drought, normalized by the mean yield across all genotypes in the trial) which was shown to be significantly different between genotypes. Genotype x environment interactions have been shown to significantly affect sugar beet yield (Hoffmann et al., 2009) and phenotypic differences related to drought tolerance, which may be observed when water is freely available, are not always as evident under drought (Ober et al., 2004). This highlights that traits must be tested under a range of conditions to fully understand how they may influence sugar beet WUE. The absence of distinctive stress sensitive developmental stages such as anthesis in the annual sugar beet crop cycle means that the relationship between water use and yield is consistent regardless of drought timing (Dunham, 1993). Indirect canopy traits have a strong influence on DTI with greater green canopy maintenance, low wilting and senescence score, specific leaf weight (DW/total sampled leaf area) and succulence index (FW-DW/total sampled leaf area) all enhancing DTI (Ober et al., 2005). WUE assessed at the crop level shows significant differences between genotypes, which is driven by increased biomass accumulation rather than reduced water use (Ober et al., 2005). There was no assessment in these studies of WUE_i_ and associated traits, such as Δ^13^C, and whether these could be correlated with DTI. Δ^13^C is strongly correlated with WUE_i_ and provides an integrated measure of WUE_i_ over time. This makes it more reliable than direct leaf gas exchange measurements, which can be influenced by the environment at the time of measurement.

Few studies have considered Δ^13^C in sugar beet but Rajabi et al. (2008) found that a greater SLW was related to an increased Δ^13^C and WUE_i_ in breeding lines and hybrids. SLW was also correlated with DTI, suggesting that there may be a relationship between drought tolerance traits and WUE. Additionally, it has been shown that variation in Δ^13^C is greater in conditions where water is not limiting as opposed to under drought (Rytter, 2005; Rajabi et al., 2009). This shows the importance of assessing Δ^13^C under both irrigated and droughted conditions to ensure that the relationship with WUE_i_ is understood. Sugar beet Δ^13^C has also been used to show the increase of WUE_i_ under drought as stomatal aperture is reduced. The decline in Δ^13^C between well-watered and droughted treatments equated to a 24% increase in WUE, and the same study highlighted that leaf Δ^13^C in sugar beet is a better measure of WUE than root Δ^13^C (Bloch et al., 2006).

Studies have shown that there is a range of drought tolerance and Δ^13^C values in sugar beet breeding lines. However, the relationship between WUE_i_ and traits associated with drought tolerance has not been explored. The consistency of SLW in both increased DTI and Δ^13^C suggest that canopy traits are closely associated with sugar beet WUE_i_ and should be a key area of focus. In cereal crops it has been shown that canopy architecture can also affect Δ^13^C with more erect leaves having a greater Δ^13^C and achieving greater yields but the effect on WUE was not assessed (Araus et al., 1993). Sugar beet canopies can be classed as upright or prostrate and research has shown that this drives differences in radiation use efficiency (RUE) (Tillier et al., 2023). Varieties with upright canopies were shown to have a higher RUE than the prostrate, potentially driven by the leaves being better able to intercept light later in the year as the sun is lower in the sky. As radiation drives transpiration as well as photosynthesis this could lead to differences in WUE between upright and prostrate varieties which is yet to be studied. It could be hypothesized that the prostrate variety would have a greater WUE because less leaves in the canopy are exposed to high levels of radiation reducing transpiration. However, this could come at a cost to yield as overall less light is intercepted by the canopy for photosynthesis. Additionally, the differences in canopy architecture could also drive differences in leaf physiology as leaves could be different in structure to enable them to sit in a more upright or prostrate position. They could also differ in stomatal physiology as the abaxial surface of a leaf in an upright canopy is more likely to intercept more light than in a prostrate variety. Previous work has been focused on the Beta genebank and breeders’ lines rather than elite commercial varieties. So far, differences in traits associated with greater WUE_i_ have not been detected in elite varieties which, if identified, would show that greater WUE_i_ in sugar beet is a commercially viable trait for breeders to target. Although work is being undertaken to identify traits in wild sea beet populations, which may be introgressed into commercial varieties (Ribeiro et al., 2016), progress is slow as it relies on traditional breeding techniques. Therefore, it is useful to explore whether differences in WUE_i_ and associated traits are evident in commercial varieties. If differences are identified, this would show that increased WUE_i_ is already a viable trait in commercial sugar beet crops. Therefore, in this study, two elite UK sugar beet varieties, with contrasting upright and prostrate canopies, have been selected to answer the research questions:

1. How is WUE affected by fluctuations in soil water availability in sugar beet?
2. Does sugar beet acclimate to water deficit by increasing WUE?
3. Are there differences in WUE between commercial sugar beet varieties with contrasting canopy architecture, and if so, is this related to stomatal morphology and leaf traits such as SLW and RWC?

## 2 Materials and methods

### 2.1 Box set up and plant materials

**Figure 1:**
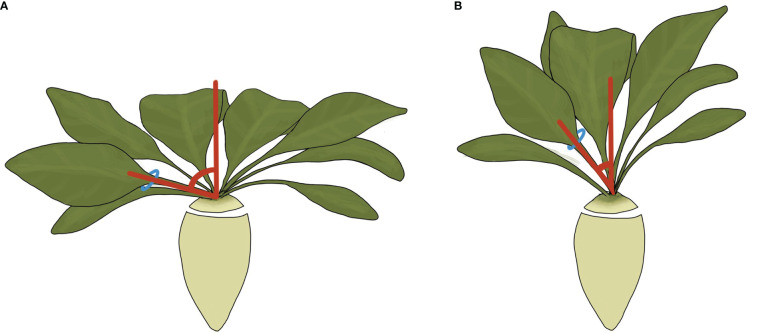
An illustration of a prostrate **(A)** and upright **(B)** sugar beet canopy. The petiole angle is shown in red and is greater in prostate varieties. Originally published in Tillier et al. (2023); reproduced under CC BY 4.0.

Two experiments were conducted in 2018 and 2019 using sugar beet varieties from different breeders with contrasting canopies (**Figure 1**), Cayman with a prostrate canopy (Prostrate) and Sabatina KWS with an upright canopy (Upright). To simulate a realistic canopy environment sugar beet were grown in plastic pallet boxes with a volume of 610 L, depth of 60 cm and surface area of 1.1 m^2^. Boxes had drainage holes drilled in the bottom with membrane overlaid and filled with a sandy clay loam, (Landscape20, Topsoil, Cambridgeshire, UK). The boxes were filled in four stages, with a minimum of 6 days between each stage, and hand watered to settle the soil. Volumetric water sensors (ECH_2_0 EC-5, Meter group Inc, USA) were buried 15 cm from the bottom of the box in 2018 whilst in 2019 larger sensors (ECH_2_0 10HS, Meter group Inc, USA) were buried at 30 cm to get a reading over a larger soil volume. Sensors were calibrated specifically to the soil used, as directed by the manufacturer’s protocol. Volumetric water content (VWC) was logged every hour (Em5b, Meter group Inc, USA). The boxes were placed in an open ended polytunnel without environmental controls orientated East to West and covered in a diffuse polythene (SunMaster Diffused, XL Horticulture LTD, Devon, UK) in 2018 and a clear polythene (SunMaster Clear, XL Horticulture LTD, Devon, UK) in 2019. In both years, the boxes were arranged in a split plot design, with irrigation regime on the main plot and variety on the sub plot. Measurements were taken on 32 boxes divided into four blocks of eight with discard boxes at the end of each row to minimise edge effects. A temperature and humidity sensor was suspended at canopy height and logged measurements every hour (TinyTag Ultra 2, Chichester, UK). In 2019, an additional sensor was suspended at the end of blocks 2 and 4 to identify if a temperature gradient was present but no differences were identified. Thermal time was higher in 2019 than 2018 with differences most evident in August and September (**Supplementary Figure S1**).

### 2.2 Sowing and establishment

To ensure an optimal seedbed the boxes were raked to produce a fine tilth. A plywood board with holes drilled for correct seed spacing (**Supplementary Figure S2**) was placed over the box and three seeds sown in each hole. Boxes were hand watered at regular intervals, and timed to ensure equal watering, to prevent soil drying and ensure good establishment. Plants were thinned once two true leaves were evident to give a total of 12 plants per box. Boxes were fertilised with ammonium nitrate in 2018 and ammonium sulphate in 2019, using a split application equating to 40 kg N ha^-1^ followed by 80 kg N ha^-1^. After each application of fertiliser, the boxes were watered equally. In both years the seeds were sown on 9 April, in 2018 fertiliser was applied at 15 and 29 DAS with thinning at 32 DAS, whilst in 2019 fertiliser was applied at 16 and 29 DAS with thinning at 29 DAS.

### 2.3 Irrigation

An irrigation system consisting of drip irrigation pipe was installed after emergence with three lengths of pipe running between the sugar beet rows (**Supplementary Figure S2**). Four irrigation treatments were applied; a fully irrigated control (Full), a single drought (SD), a double drought (DD) which had the SD treatment plus a second period of drought and a water limited treatment (Ltd) which was kept at approximately 50% field capacity. The second water withdrawal for the DD started when the maximum rate of assimilation (A_max_) returned to a level similar to the fully irrigated plants. Boxes were irrigated back to field capacity immediately at the end of a water withdrawal period. Timings were comparable between the two years, except for the DD which was later in 2018 than 2019. The first water withdrawal was at 65-96 DAS in 2018 and 73-92 DAS in 2019 and the second at 151-200 DAS in 2018 and 129-148 DAS in 2019. In 2018, the total amount of water applied per box was; Full – 110.9 L, SD- 102.1 L, DD-75.9 L and Ltd- 41.3 L and in 2019; Full – 102 L, SD- 90.1 L, DD-83.6 L and Ltd- 28.9 L. Soil moisture was monitored using the VWC sensors in 2018 and 2019 (**Supplementary Figures S3A, B**) and irrigation adjusted accordingly.

### 2.4 Leaf gas exchange and chlorophyll fluorescence spot measurements

Spot measurements of gas exchange measurements of maximum assimilation (A_max_) and stomatal conductance (g_s_) were taken using an infra-red gas analyser (LiCor Li6800, LiCor, USA) and used to calculate WUE_i_ (Condon et al., 2002).


Equation 1 $$WUE_{i}=\;\frac{A}{gs}$$

The two-sugar beet located centrally in the box (6 and 7, **Supplementary Figure S2**) were measured to avoid any potential edge effects. Settings were: flow 500 µmol s⁻¹, heat exchanger temperature 20°C (which gave a leaf temperature between 20°C and 28°C dependent on ambient conditions), RH 50%, light 1200 µmol m⁻² s⁻¹, and matched every 10 minutes. Chlorophyll fluorescence parameters of F_v_’/F_m_’ (maximum PSII efficiency in the light), ΦPSII (quantum efficiency of PSII electron transport in the light) and q_p_ (photochemical quenching) were measured at the same time using a multiphase flash fluorometer (LiCor Li6800, LiCor, USA) and a dark pulse (Murchie and Lawson, 2013). Data was logged once A, g_s_, CO_2_ sample and H_2_O sample were stable, which took between 5-10 minutes per leaf. Two leaves from each plant, referred to as ‘measurement leaf 1’ and ‘measurement leaf 2’ were focused on each year. Measurements started once the leaves were fully expanded with measurement leaf 1 used from 77-133 DAS in 2018 (includes the first drought from 65-96 DAS) and in 2019 this leaf was used from 69-110 DAS (includes the drought at 73-92 DAS). Measurement leaf 2 was used from 105-210 DAS in 2018, (second drought 151-200 DAS) and from 118-182 DAS in 2019 (second drought 129-148 DAS).

In 2019 measurements were also taken of ‘measurement leaf 3’ at 162-182 DAS to correlate with Δ^13^C, which will be outlined later. Measurements were taken between 8:00 hr and 14:00 hr over 2 consecutive days with blocks one and two measured on the first day and blocks three and four on the second. The DAS of the first day of measurements is used to denote the timing of the measurement. When gas exchange measurements were completed, canopy temperature was assessed using thermal images of each box taken at a distance of 1 metre perpendicular to the edge of the box with a handheld camera (FLIR C2 thermal imaging camera, FLIR, USA) and analysed using thermal analysis and reporting software (FLIR Tools, FLIR, USA).

### 2.5 Chlorophyll extraction

In 2019, chlorophyll extraction was undertaken on leaf discs collected at 203 DAS from plants one, two and three (**Supplementary Figure S2**). Each leaf disc was wrapped in aluminium foil and placed in liquid nitrogen before storage in a -80°C freezer. At the same time three additional leaf discs were cut from each leaf and weighed together to determine FW and oven dried to record DW.

The frozen leaf discs were added to 2 ml microcentrifuge tubes with 1.5 ml 80% acetone and a ceramic bead before milling using a fast prep for two 20 sec cycles. The extracted chlorophyll was then transferred to 15 ml centrifuge tubes and topped up to 4 ml using 3 ml 80% acetone before centrifuging for 5 min at 3000 rpm. Chlorophyll *a* and *b* concentrations were then measured using a spectrophotometer (Cary 50, Agilent, Santa Clara, US) using the method of Porra (2002).

### 2.6 Relative water content

Relative water content (RWC) was measured at regular intervals from 66-213 DAS in 2018 and 74-177 DAS in 2019. Using a cork borer three 1 cm diameter leaf discs were cut from a leaf on plants 2 and 3 representative of the new measurement leaf on plants 6 and 7 (**Supplementary Figure S2**). The 6 leaf discs were processed using the method of Weatherley (1950) and RWC calculated using the following equation:


Equation 2 $$RWC=\;\frac{\left(FW-DW\right)}{\left(TW-DW\right)}\;\times \;100$$

### 2.7 Stomatal impressions

Stomatal impressions of the abaxial and adaxial leaf surface of leaves of a similar age were taken at 219 DAS and 203 DAS in 2018 and 2019 respectively. Clear nail varnish was applied and left to dry for 20 minutes until no longer tacky, lifted using clear tape and mounted on a microscope sample slide. Three images were taken from each sample slide using a microscope (Leica 5000B, Wetzlar, Germany) with a light source (Leica CTR5000 Wetzlar, Germany) at 100x magnification and cropped to 1 mm^2^ using the microscope scale for reference in Fiji (Schindelin et al., 2012). The stomata in the cropped images were manually counted in Fiji using the Cell Counter plug in (Author: Kurt De Vos), with an average stomatal density (SD) for each sample calculated from the three 1 mm^2^ areas.

### 2.8 Harvest

Boxes were harvested at 226 DAS and 211 DAS in 2018 and 2019 respectively. The sugar beet were hand lifted with plants 6 and 7 taken for further analysis. The 10 remaining beet were topped and the leaves and roots weighed separately to determine FW. The canopy was then discarded, and the roots taken to the BBRO tare house at Wissington Sugar Beet factory, Norfolk, UK to determine sugar % using polarimetry. K and Na impurities were also measured using flame photometry and AN content using colorimetry as per standard methods (ICUMSA, 2022). The leaves and roots of plants 6 and 7 were combined and weighed to determine FW before oven drying at 70°C and weighed to determine leaf and root dry matter (DM). The %DM of leaves and roots from plants 6 and 7 in each box was used to calculate the total DM from the total FW. The white sugar yield (WS) was calculated by multiplying the total FW by the sugar percentage. The total DM and WS for each box and the total amount of irrigation applied was then used to calculate the box level total dry matter water use efficiency (WUE_DM_) and WS water use efficiency (WUE_WS_).


Equation 3 $$WUE_{DM}=\;\frac{Box\;total\;DM}{Total\;volume\;of\;water\;applied\;per\;box\;}\;$$

Equation 4 $$WUE_{WS}=\;\frac{Box\;total\;WS\;}{Total\;volume\;of\;water\;applied\;per\;box\;}\;$$

### 2.9 Specific leaf weight

Specific leaf weight (SLW) was calculated from measurement leaf 3, before processing to determine Δ^13^C, with the leaf passed through a leaf area meter (Li-3100, LiCor, USA) to determine leaf area with DW calculated from the FW multiplied by the %DW derived from beet 6 and 7 at harvest.


Equation 5 $$SLW=\;\frac{DW\;}{Area\;}$$

### 2.10 Carbon 13 isotope discrimination

In 2019, measurement leaf 2 and 3 were removed at 209 DAS and freeze dried to determine the ration of ^12^C to ^13^C (δ13C). Samples were milled (ZM200, RETSCH, Haan, Germany) to a fine homogenised powder and analysed at the British Geological Survey in Keyworth, Nottinghamshire. Leaf δ^13^C analyses were performed by combustion in a Costech Elemental Analyser (EA) on-line to a VG TripleTrap and Optima dual-inlet mass spectrometer, with δ13C values calculated to the VPDB scale using a within-run laboratory standards calibrated against NBS 18, NBS 19 and NBS 22. Replicate analysis of well-mixed samples indicated a precision of ±<0.1‰ (1 SD). δ^13^C was used to calculate carbon isotope discrimination (Δ^13^C), which is inversely proportional to WUE.


Equation 6 $${\text{Δ}}^{13}C=\frac{{\text{δ }}_{a}-{\text{δ }}_{p}}{1+{\text{ δ }}_{p}}\;$$

Where δ_p_ is the δ13C calculated from the leaf tissue and δ_a_ is the atmospheric ratio of ^12^C to ^13^C taken to be -8‰ Δ^13^C (Farquhar et al., 1989).

Δ^13^C was plotted against average WUE_i_ on a per leaf basis, (calculated from the gas exchange values taken from measurement leaf 2 and 3), and WUE_DM_, WUE_WS_ and SLW using averages calculated on a per box basis.

### 2.11 Statistical analysis

Repeated measures ANOVA for a split plot design was conducted on the A_max_, g_s_, C_i_/C_a_, WUE_i_, F_v_/’F_m_’, ΦPSII, q_p_, canopy temperature and RWC data. There were four blocks and whole plots dictated by the irrigation regime with variety and irrigation as factors. For the total leaf chlorophyll content, leaf water content, stomatal density, WS yield, Total DM, WUE_WS_, WUE_DM_, SLW and Δ^13^C data a general split plot ANOVA with the same factors as the repeated measures ANOVA was used. All analysis was undertaken in GenStat 19^th^ edition (VSN International Ltd., Hemel Hempstead, United Kingdom).

## 3 Results

### 3.1 Leaf gas exchange and WUE_i_

**Figure 2:**
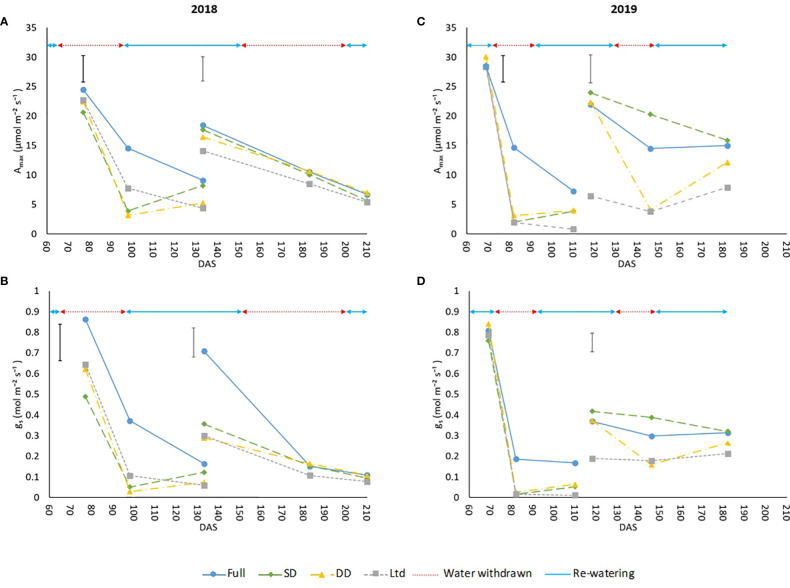
The maximum assimilation rate (A_max_) and stomatal conductance (g_s_) of sugar beet grown under four irrigation regimes, measured using an infra-red gas analyser (Li6800, LiCor, Nebraska, US). Measurements were taken from 2 leaves Measurement leaf 1 covers the first drought and measurement leaf 2 the second drought in 2018 and 2019. **(A)** leaf 1 (LSD=4.54 DF=190 P<0.001), leaf 2 (LSD=4.16 DF=191 P=0.002). **(B)** leaf 1 (LSD=0.177 DF=190 P=0.005), leaf 2 (LSD=0.141 DF=191 P=0.002). **(C)** leaf 1 (LSD=4.45 DF=220 P<0.001), leaf 2 (LSD=4.75 DF=255 P<0.001). **(D)** leaf 1 (P=0.133), leaf 2 (LSD=0.136 DF=255 P<0.001). Error bars show time x irrigation LSD for all data points. ANOVA tables containing all the data points are available for A_max_ and g_s_ in **Supplementary Table S5** (2018) and **Table S6** (2019). Irrigation regimes were a fully irrigated (Full), a continually water limited kept at approx. 50% field capacity (Ltd), a single drought (SD) (2018 65-96 DAS and 2019 73 -92 DAS) and a double drought (DD) which was exposed to the single drought treatment plus an additional drought (2018 151-200 DAS and 2019 118-182 DAS).

No leaf gas exchange interactions were observed between water availability x variety in the spot measurements, so the data was averaged across the two sugar beet varieties to examine the effect of water availability on WUE. Differences in some of the gas exchange parameters were observed in 2019 between the varieties and in Δ^13^C, which gives a longer-term measure of WUE_i_, and are presented later. A_max_ and g_s_ were significantly reduced in all treatments over time as leaves aged (**Figure 2**). Drought reduced A_max_ and g_s_ compared to the fully irrigated plants 92 DAS, 23 days after water withdrawal in 2018 (P<0.001; **Figures 2A, B**), in 2019 A_max_ was reduced 81 DAS, only 9 days after water withdrawal (P<0.001; **Figure 2C**) and g_s_ was reduced, but not significantly, compared to the fully irrigated (**Figure 2D**). In 2018 the slight reduction in A_max_ and g_s_ in the DD treatment compared to the fully irrigated at 196 DAS (**Figures 2A, B**) may be attributed to the drought being later in the year when the lower temperature reduced transpiration, as shown by the lower thermal time during the second drought in 2018 compared to 2019 (**Supplementary Figure S1**). During the second drought in 2019, A_max_ and g_s_ were reduced in the DD treatment (P<0.001; **Figures 2C, D**) at 140 DAS, 11 days after water withdrawal. At 140 and 146 DAS the SD treatment had a higher A_max_ and g_s_ than the fully irrigated (P<0.001; **Figures 2C, D**). During this period air temperatures exceeded 40°C from 136 – 140 DAS and the fully irrigated treatment saw a decline in VWC to levels similar to the water limited treatment before additional irrigation was applied at 141 and 142 DAS with this difference no longer evident at 153 DAS.

Recovery from the first drought, when A_max_ was no longer significantly different to the control, in 2018 was at 133 DAS, 37 days after rewatering (**Figure 2A**), and in 2019 was at 109 DAS, 18 days after rewatering (**Figure 2C**). Due to the lack of significant decline in A and g_s_ under the second drought in 2018, recovery was only measured in 2019 and this was evidenced at 174 DAS, 46 days after rewatering. The close relationship between A_max_ and g_s_ means that g_s_ showed the same trends in recovery as A_max_. The decline and recovery of A_max_ is further supported by the changes in chlorophyll fluorescence parameters (**Supplementary Figures S4A–D**) of maximum photochemical efficiency in the light, F_v_/’F_m_’ (**Figures S4A, D**), PSII operating efficiency, ΦPSII (**Figures S4B, E**), and photochemical quenching, q_p_ (**Figures S4C, F**), which are largely consistent with those expected from a change in A_max_ as a result of stomatal closure.

The continually water limited treatment had a reduced A_max_ and g_s_ comparable to the droughted treatments during water withdrawal, with the slower reduction in VWC in 2018 (**Figures 2A, B**) meaning the decline in A_max_ and g_s_ was not as rapid as it was in 2019 (**Figures 2C, D**). Once VWC was maintained at approximately 50% field capacity A_max_ and g_s_ were lower in the water limited treatment compared to the fully irrigated throughout the measurement periods in both years (P<0.001; **Figure 2**), except from 183 DAS onwards in 2018 (**Figures 2A, B**), and g_s_ at 133 DAS as the fully irrigated leaf g_s_ had also declined (**Figure 2B**).

**Figure 3:**
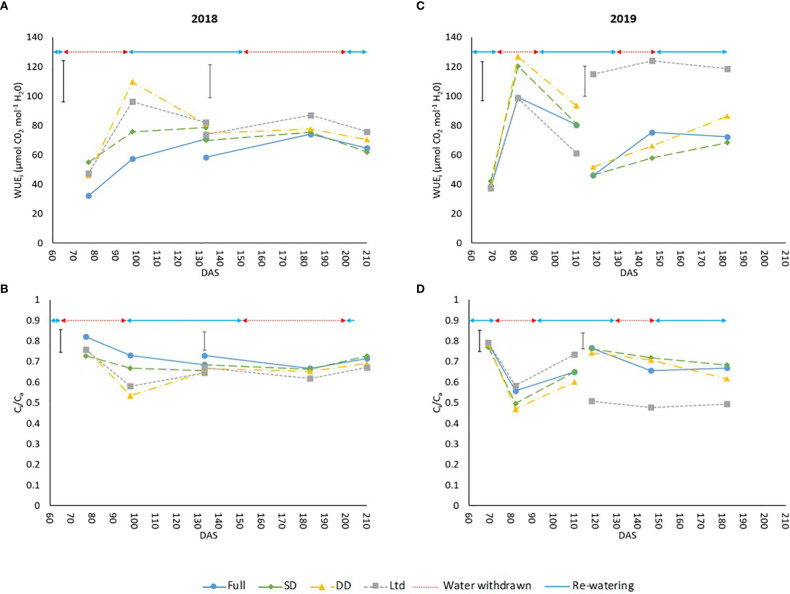
The intrinsic water use efficiency (WUE_i_) and ratio of intercellular to ambient CO_2_ (Ci/C_a_) of sugar beet grown under four irrigation regimes, measured using an infra-red gas analyser (Li6800, LiCor, Nebraska, US). Measurements were taken from 2 leaves Measurement leaf 1 covers the first drought and measurement leaf 2 the second drought in 2018 and 2019. **(A)** leaf 1 (LSD=28.2 DF=190 P=0.004), leaf 2 (LSD=22.4 DF=191 P=0.004). **(B)** leaf 1 (LSD=0.110 DF=190 P=0.006), leaf 2 (LSD=0.088 DF=191 P=0.004). **(C)** leaf 1 (LSD=26.5 DF=220 P=0.003), leaf 2 (LSD=20.4 DF=255 P=0.022). **(D)** leaf 1 (LSD=0.104 DF=220 P=0.003), leaf 2 (LSD=0.079 DF=255 P=0.027). Error bars show time x irrigation LSD for all data points. ANOVA tables containing all the data points are available for WUE_i_ and C_i_/C_a_ in **Supplementary Table S5** (2018) and **Table S6** (2019). Irrigation regimes were a fully irrigated (Full), a continually water limited kept at approx. 50% field capacity (Ltd), a single drought (SD) (2018 65-96 DAS and 2019 73 -92 DAS) and a double drought (DD) which was exposed to the single drought treatment plus an additional drought (2018 151-200 DAS and 2019 118-182 DAS).

WUE_i_ was greater in the drought treatments of measurement leaf 1 in 2018 with all treatments having a higher WUE_i_ than the fully irrigated until 133 DAS (P=0.004; **Figure 3A**), which could be attributed to a lower relative decline of A_max_ and g_s_ in comparison as leaves age. The C_i_/C_a_ values show a significant reduction compared the fully irrigated until 133 DAS (P=0.006; **Figure 3B**) as stomatal aperture is reduced to conserve water which is also driving the increased WUE_i_. In 2019, the difference in WUE_i_ between treatments was not consistent and from 97-110 DAS the Ltd treatment had a lower WUE_i_ than the fully irrigated, the only example of a water deficit treatment having a lower WUE_i_ than the fully irrigated (P=0.003; **Figure 3A**). This was also reflected in the C_i_/C_a_ values (**Figure 3B**). In 2018, the Ltd treatment had a higher WUE_i_ at 105 and 113 DAS in measurement leaf 2 where it covers the first drought (P=0.004; **Figure 3A**). The C_i_/C_a_ also reflects this with the Ltd treatment being lower than the SD and DD treatment which were themselves lower than the fully irrigated (P=0.004; **Figure 3B**). At 196 DAS a higher WUE_i_ was also evident in the Ltd treatment (P=0.004; **Figure 3A**) alongside a reduced C_i_/C_a_ (P=0.004; **Figure 3B**) compared to the other treatments. The DD treatment showed no increase in WUE_i_ (**Figure 3A**) but A_max_ and g_s_ were not significantly reduced as previously outlined. In 2019, the second drought increased the WUE_i_ of the DD treatment at 153 and 163 DAS with this difference no longer significant at 169 DAS (P=0.022; **Figure 3C**), just before recovery of A_max_ and g_s_ at 174 DAS. This also resulted in a decline in C_i_/C_a_ compared to the fully irrigated and SD to levels similar to the Ltd treatment (P=0.027; **Figure 3D**). The increase in WUE_i_ (P=0.022; **Figure 3C**) and decrease in C_i_/C_a_ (P=0.027; **Figure 3D**) in the fully irrigated compared to the SD at 140 and 146 DAS can be attributed to the decline in VWC previously outlined.

Averaged over all treatments at all measurement points there were no consistent differences in A_max_, g_s_, WUE_i_ and C_i_/C_a_ between varieties in 2018. In 2019 there was no significant difference in A_max_ between the two sugar beet varieties, however g_s_ was significantly higher in the upright variety of both measurement leaf 1: 0.284 µmol m⁻² s⁻¹ compared to 0.196 µmol m⁻² s⁻¹ (P=0.012) and 2: 0.279 µmol m⁻² s⁻¹ compared to 0.197 µmol m⁻² s⁻¹ (P=0.042). The non-significant difference in A_max_ between varieties coupled with a significantly lower g_s_ resulted in a trend (P=0.072) of greater WUE_i_ in the prostrate variety in measurement leaf 1 of 77.2 µmol CO_2_ mol^-1^ H_2_O compared to 70.4 µmol CO_2_ mol^-1^ H_2_O (P=0.072) and was significantly greater for measurement leaf 2 at 86.0 µmol CO_2_ mol^-1^ H_2_O compared to 75.8 µmol CO_2_ mol^-1^ H_2_O (P=0.011). This greater WUE_i_ was also associated with a lower C_i_/C_a_ for measurement leaf 2 of the prostrate variety of 0.655 compared to 0.681 (P=0.012) and a similar trend for measurement leaf 1 at 0.615 compared to 0.657 (P=0.069).

### 3.2 Canopy temperature

Canopy temperature can increase relative to air temperature as a result of reduced stomatal aperture and lowered transpiration. Looking at the effect of water availability across the two varieties during the first drought in 2018 and 2019 and the second drought in 2019 (not the second drought in 2018 due to the cool temperatures later in the season), an increase in absolute canopy temperature was evident (**Supplementary Figure S7**).

**Figure 4:**
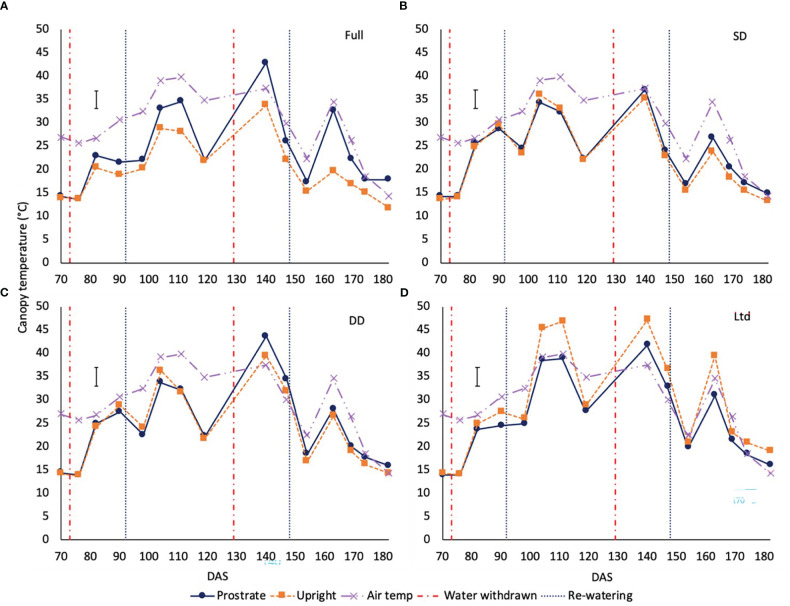
The canopy temperature of two sugar beet varieties with a prostrate and upright canopy grown under four different irrigation regimes. Error bars show variety x irrigation x time interaction (P=0.003 DF=42 LSD=3.88). Irrigation regimes were a fully irrigated (Full) **(A)**, continually water limited kept at approx. 50% field capacity (Ltd) **(B)**, a single drought (SD) **(C)** 73 -92 DAS and a double drought (DD) **(D)** which was exposed to the single drought treatment plus an additional drought 118-182 DAS.

In 2019 there was a variety x treatment x time interaction of canopy temperature. In the fully irrigated sugar beet the prostrate variety had a warmer canopy compared to the upright at 104, 111, 140, 147, 163, 174 and 182 DAS (P=0.003; **Figure 4A**). In the SD (**Figure 4B**) and DD (**Figure 4C**) treatments there were no significant differences between varieties during the first drought but during the second drought at 140 DAS the prostrate variety had a significantly warmer canopy than the upright in the DD treatment (P=0.003; **Figure 4C**), although both were above air temperature suggesting they had closed stomata. In the Ltd treatment the upright variety had a warmer canopy than the prostrate (P=0.003; **Figure 4D**), at 104, 111, 140 and 163 DAS, opposite to the observations in the fully irrigated treatment.

### 3.3 Relative water content

**Figure 5:**
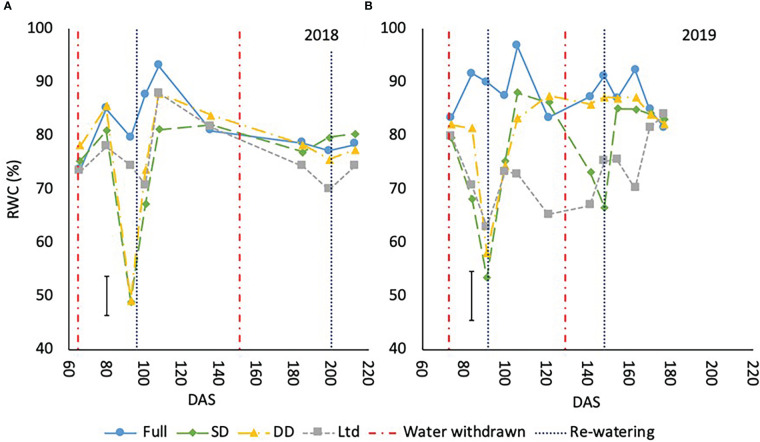
The relative water content (RWC) (%) of sugar beet grown under four different irrigation regimes in 2018 **(A)** (LSD=7.34 P<0.001) and 2019 **(B)** (LSD=9.16 P<0.001). Error bars shows irrigation x time LSD. Irrigation regimes were a fully irrigated (Full), a continually water limited kept at approx. 50% field capacity (Ltd), a single drought (SD) (2018 65-96 DAS and 2019 73 -92 DAS) and a double drought (DD) which was exposed to the single drought treatment plus an additional drought (2018 151-200 DAS and 2019 118-182 DAS).

RWC declined in the sugar beet under drought as water availability reduced. This was evident during the first drought in 2018 at 93 - 101 DAS with recovery by 108 DAS except in the SD which was lower than the fully irrigated but similar to the DD and Ltd treatments (P<0.001; **Figure 5A**). In 2019, the first drought reduced RWC from 84 – 106 DAS with recovery by 121 DAS (P<0.001; **Figure 5B**). The second drought had the same effect in 2019 reducing RWC from 141-148 DAS with recovery by 154 DAS (P<0.001; **Figure 5A**). In 2018, the late drought did not significantly reduce RWC (**Figure 5B**). The Ltd treatment had a lower RWC compared to the fully irrigated once VWC had declined from 101-108 DAS in 2018 (P<0.001; **Figure 5A**) and 84-170 DAS 2019 (P<0.001; **Figure 5B**). Despite the decline in VWC in the fully irrigated at 140-146 DAS in 2019 and the concurrent reduction in A_max_ and g_s,_ RWC did not significantly decline.

The prostrate variety had a greater RWC than the upright variety when averaged across treatments in 2018 at 81.1% compared to 73.4% (P<0.001) and in 2019 84.8% compared to 75.5% (P<0.001).

### 3.4 Leaf chlorophyll and water content

**Figure 6:**
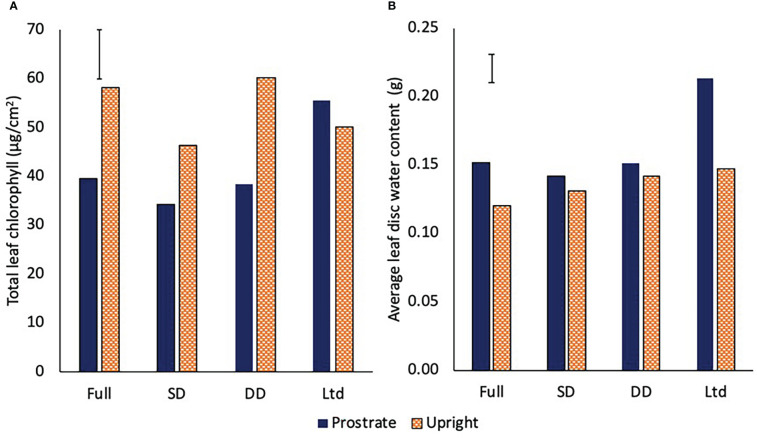
The total chlorophyll content **(A)** (P=0.008 DF=31 LSD=8.5) and leaf water content **(B)** (P=0.016 DF=31 LSD=0.021) of two sugar beet varieties with a prostrate and upright canopy grown under four different irrigation regimes, fully irrigated (Full), single drought (SD), double drought (DD) and continually water limited (Ltd). Error bar shows irrigation x variety LSD.

The prostrate variety had greater total chlorophyll content on a per unit area basis in the Ltd treatment than all the other treatments, while in the upright variety, the fully irrigated and the DD had greater chlorophyll content than the SD and the Ltd (P=0.008; **Figure 6A**). The upright variety had greater chlorophyll content than the prostrate in the fully irrigated, SD and DD treatments whilst there was no significant difference in the Ltd treatment (P=0.008; **Figure 6A**). The ratio of chlorophyll *a* to *b* was not significantly different between irrigation treatments or variety. Leaf water content was also assessed to identify if this varied with chlorophyll content but the only difference was a greater water content in the prostrate variety compared to the upright in the full and Ltd, with the Ltd water content being significantly greater than that observed in any other treatment combination (P=0.016; **Figure 6B**).

### 3.5 Stomatal density

**Figure 7:**
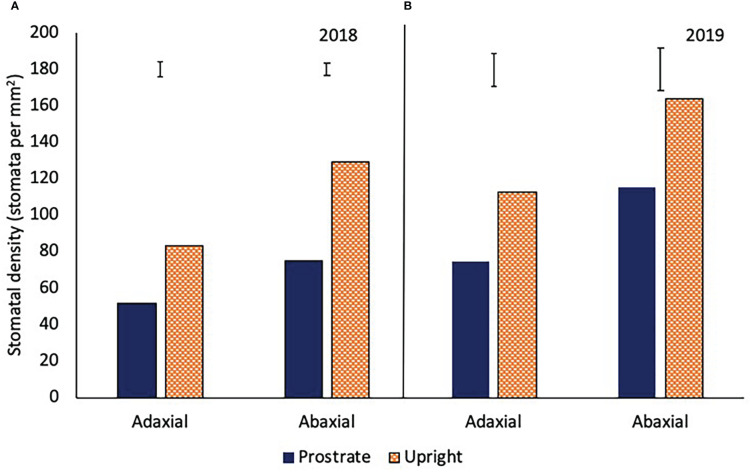
The stomatal density of the adaxial and abaxial leaves of two sugar beet varieties with a prostrate and upright canopy in 2018 **(A)** (adaxial LSD=7.7 DF=31 P<0.001, abaxial LSD=6.7 DF=31 P<0.001) and 2019 **(B)** (adaxial LSD=18.1 DF=31 P<0.001, abaxial LSD=23.1 DF=31 P<0.001). Sugar beet were grown under four different irrigation regimes, fully irrigated (Full), single drought (SD), double drought (DD) and continually water limited (Ltd) and averages over all the treatmetns are shown as there was no irrigation x variety interaction.

The prostrate variety had a significantly lower stomatal density on both the adaxial and abaxial leaf surface in 2018 and 2019 (P<0.001; **Figure 7**). There was no consistent relationship between stomatal density and irrgation in either year.

### 3.6 Yield and WUE of dry matter and white sugar yield and impurities

**Figure 8:**
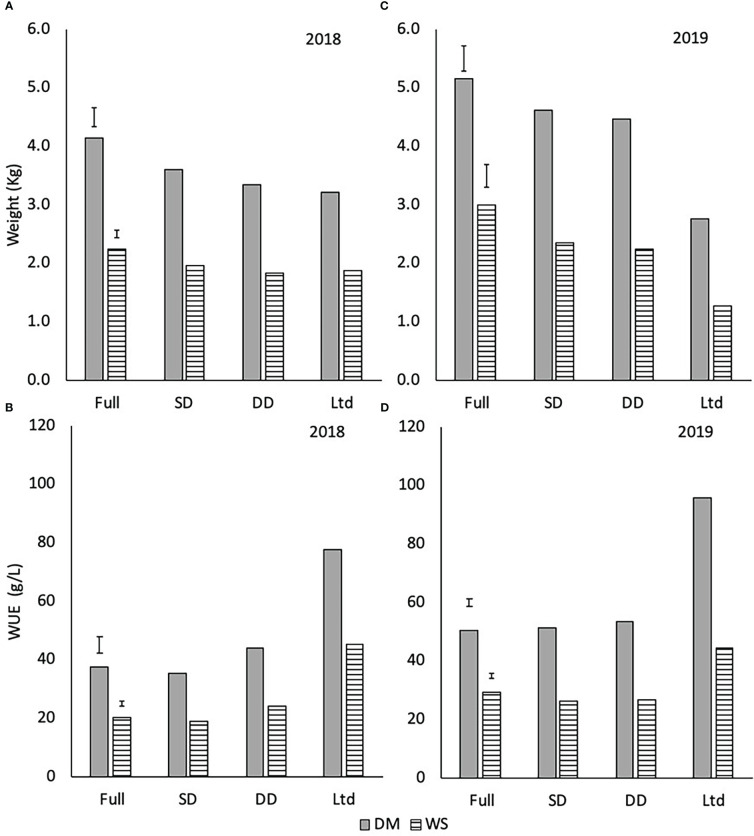
The amount of total dry matter (DM) and white sugar yield (WS) in 2018 **(A)** and 2019 **(B)** and dry matter water use efficiency (WUE_DM_) and white sugar yield water use efficiency (WUE_WS_) in 2018 **(C)** and 2019 **(D)** of sugar beet grown under four different irrigation regimes. **(A)** 2018 weights (DM LSD=0.322 DF=31 P<0.001 and WS LSD=0.142 DF=31 P<0.001). **(B)** 2019 weights (DM LSD=0.222 DF=31 P<0.001 and WS LSD=0.192 DF=31 P<0.001). **(C)** 2018 WUE (WUE_DM_ LSD=5.76 DF=31 P<0.001 and WUE_WS_ LSD=1.78 DF=31 P<0.001). **(D)** 2019 WUE (WUE_DM_ LSD=2.52 DF=31 P<0.001, WUE_WS_ LSD=2.09 DF=31 P<0.001). Error bars show irrigation LSD. Irrigation regimes were a fully irrigated (Full), a continually water limited kept at approx. 50% field capacity (Ltd), a single drought (SD) (2018 65-96 DAS and 2019 73 -92 DAS) and a double drought (DD) which was exposed to the single drought treatment plus an additional drought (2018 151-200 DAS and 2019 118-182 DAS).

There were no varietal differences in the total dry matter or white sugar yields and the WUE_DM_ and WUE_WS_ so these observations were averaged across the two varieties to focus on the effect of water availability. The fully irrigated treatment had a greater total DM and WS yield in 2018 (P<0.001; **Figure 8A**) and 2019 (P<0.001; **Figure 8B**) than the other three treatments. In 2019, the Ltd treatment resulted in lower total DM and WS than the SD and DD (P<0.001; **Figure 8B**). In 2019 the sugar beet achieved a higher total DM and WS than 2018, except in the Ltd treatment, highlighting the differences in the crop’s growth between years.

The WUE_DM_ and WUE_WS_ was higher in the Ltd treatment than the other three treatments and nearly double that of the fully irrigated and SD treatments in 2018 (P<0.001; **Figure 8C**). The DD had a higher WUE_DM_ and WUE_WS_ compared with the fully irrigated and SD in 2018 (P<0.001; **Figure 8C**), despite having a similar total DM and WS. In 2019, the WUE_DM_ of the Ltd treatment was higher than the other three treatments (**Figure 8D**; P<0.001). The WUE_DM_ of the fully irrigated, SD and DD were similar but the WUE_WS_ in the SD and DD was lower than the fully irrigated (**Figure 8D**; P<0.001). Overall, the extreme water deficit of the Ltd treatment increased WUE but the SD and DD treatments had inconsistent effects.

There were no differences in impurities (K, Na and AN) between the irrigation treatments in 2018. In 2019 impurities significantly increased in the SD, DD and Ltd treatments compared to the irrigated control (**Supplementary Figure S8**). The SD and DD had similar levels of K whilst NA was significantly (P<0.001) higher in the DD treatment than the SD and Ltd treatments. AN levels increased with declining water availability. There were also differences in impurities between the varieties (**Supplementary Figure S9**) with the upright variety having significantly higher K (P<0.001) and Na (P<0.001) than the prostrate whilst AN levels were similar.

### 3.7 Carbon 13 isotope discrimination

**Figure 9:**
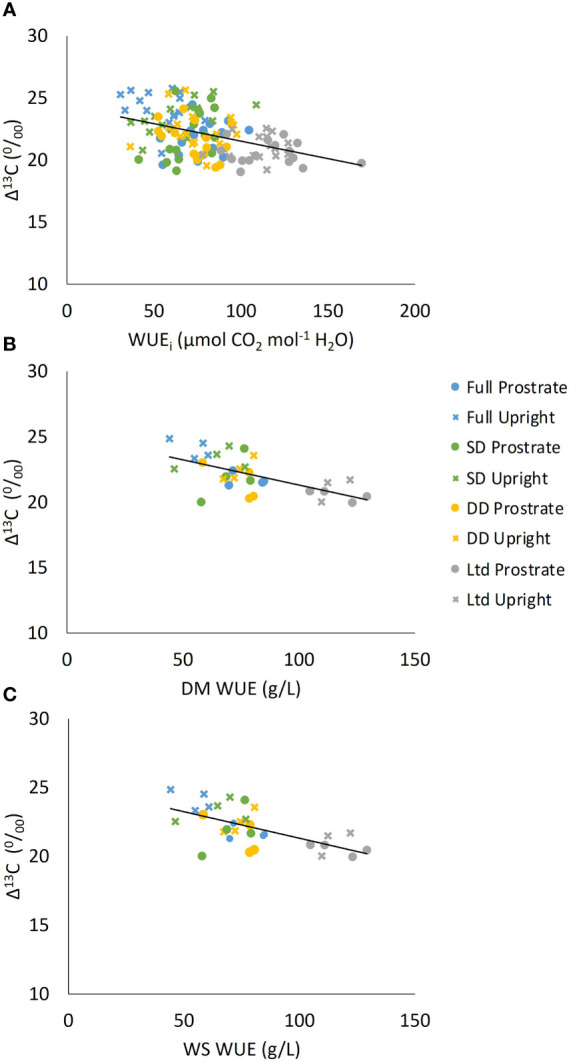
The relationship between carbon isotope discrimination (Δ^13^C) and **(A)** intrinsic water use efficiency (WUE_i_) (P<0.001 R^2 =^ 0.17), **(B)** Dry matter water use efficiency (WUE_DM_) (P=0.001 R^2 =^ 0.30) and **(C)** White sugar yield water use efficiency (WUE_WS_) (P=0.004 R^2 =^ 0.24). Two sugar beet varieties were grown under four different irrigation regimes and are plotted as one data set but presented so that the different varieties and treatments can be identified.

Δ^13^C was negatively related to the average WUE_i_ (P<0.001; **Figure 9A**), WUE_DM_ (P=0.001) (**Figure 9B**) and WUE_WS_ (P=0.004) (**Figure 9C**).

**Figure 10:**
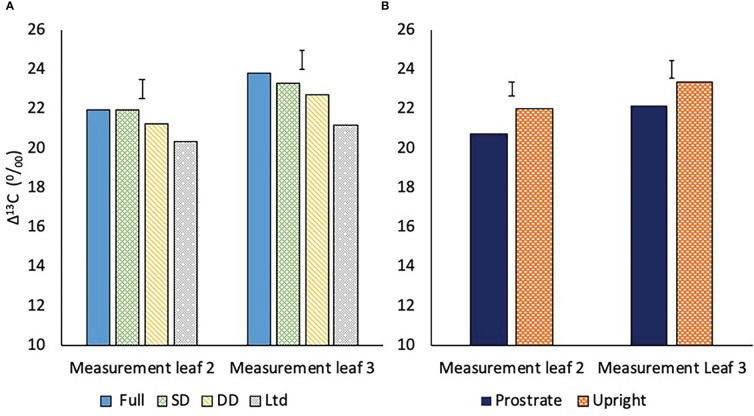
**(A)** The carbon isotope discrimination (Δ^13^C) of two measurement leaves of sugar beet grown under four different irrigation regimes, fully irrigated (Full), single drought (SD), double drought (DD) and continually water limited (Ltd). Error bar shows irrigation LSD of measurement leaf 2 (P=0.016 DF=31 LSD=0.975) and measurement leaf 3 (P=0.001 DF=31 LSD=0.984). **(B)** The Δ^13^C of two sugar beet varieties with a prostrate and upright canopy averaged across four watering regimes. Error bar shows variety LSD of measurement leaf 2 (P=0.001 DF=31 LSD=0.701) and measurement leaf 3 (P=0.012 DF=31 LSD=0.909).

Water deficit reduced Δ^13^C indicating increased WUE_i_. The Δ^13^C of measurement leaf 2, which was fully expanded at 118 DAS; before the second drought, was similar in the fully irrigated and SD treatments, with the DD lower, but not significantly (**Figure 10A**). The Ltd treatment had a lower Δ^13^C than the fully irrigated and SD but was not different to the DD treatment (**Figure 10A**; P=0.016).

Measurement leaf 3 was fully expanded at 162 DAS; after the second drought, with Δ^13^C generally higher than in measurement leaf 2 (**Figure 10A**). This can be attributed to younger leaves being more active with higher transpiration, as shown by the gas exchange measurements, resulting in reduced WUE_i_. The Δ^13^C of measurement leaf 3 was lower in the DD than the fully irrigated and the difference was significant, unlike in measurement leaf 2, showing that water deficit was reducing Δ^13^C and hence increasing WUE_i_ at a greater magnitude in younger than older leaves (**Figure 10A**; P=0.016). The SD remained similar to the fully irrigated and the Ltd treatment Δ^13^C was lower than the fully irrigated and SD but not the DD (**Figure 10A**; P=0.016).

Averaged across the watering regimes, the prostrate variety had a lower Δ^13^C than the upright in both measurement leaf 2 (P=0.001) and measurement leaf 3 (P=0.012) (**Figure 10B**).

### 3.8 Specific leaf weight

**Figure 11:**
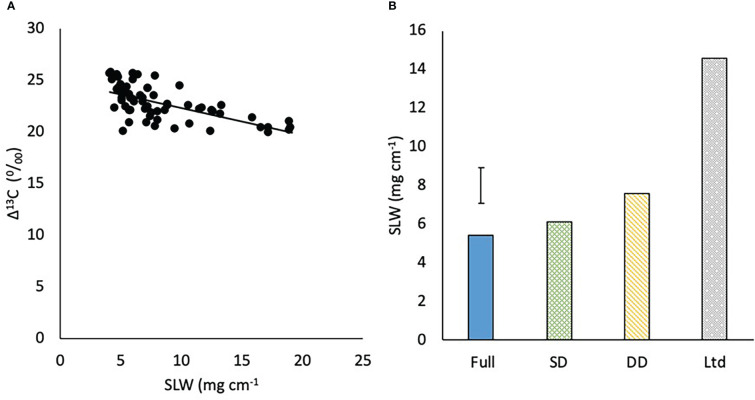
**(A)** The relationship between carbon isotope discrimination (Δ^13^C) and specific leaf weight (SLW) (P<0.001 R^2 =^ 0.41) and **(B)** the specific leaf weight (SLW) of sugar beet grown under four different irrigation regimes, fully irrigated (Full), single drought (SD), double drought (DD) and continually water limited (Ltd). Error bar shows irrigation LSD (P=0.016 DF=31 LSD=1.83).

Δ^13^C was negatively related to SLW (P=0.001; **Figure 11A**). There was no significant difference in SLW between the varieties but high levels of water deficit increased SLW with the DD having a greater SLW than the fully irrigated and SD (P=0.016; **Figure 11B**). The SLW of the Ltd treatment was almost double the next nearest SLW value in the DD.

## 4 Discussion

The relationship between Δ^13^C, dry matter accumulation and WUE_DM_ has previously been evidenced in sugar beet in response to drought (Rytter, 2005; Bloch et al., 2006). The difference between the levels of Δ^13^C in dry matter and soluble sugars (Monti et al., 2006) and leaf and root tissue (Bloch et al., 2006) have also been compared. However, this is the first time a difference in Δ^13^C has been identified in commercial sugar beet varieties and the relationship between WS yield and Δ^13^C demonstrated. Differences in WUE_i_ between the varieties were not consistently observed in the spot measurements but Δ^13^C provides a measure of WUE_i_ over a prolonged period of time so is a more reliable measure (Mairata et al., 2022). This shows that the objective of developing sugar beet varieties that are more efficient in their water use, without detriment to yield, is viable. The underlying plant metabolism and regulatory mechanisms were not studied and could be explored in a further study. This is because this study was aimed at producing information for breeders who use physiological traits for selection of sugar beet breeding lines in the field.

### 4.1 How is WUE affected by fluctuations in soil water availability in sugar beet?

Increased WUE_i_ under water deficit, as evident in our results, has been shown previously in sugar beet (Bloch et al., 2006; Rinaldi and Vonella, 2006; Fitters et al., 2018). As the water deficit increased the stomata began to close to conserve water and g_s_ declined with the reduction in transpiration causing an increased canopy temperature (Baker et al., 2007). The reduction in stomatal aperture also reduced C_i/_C_a_ which is associated with a greater WUE_i_. This in turn is related to a lower Δ^13^C as the CO_2_ in the sub stomatal cavity is not replenished as readily leading to greater a proportion of ^13^C being fixed, thereby lowering the ratio of ^13^C to ^12^C (Seibt et al., 2008). There were two exceptions to this observation. Firstly in 2019 the fully irrigated, SD and DD had similar WUE_DM_ but the fully irrigated had a higher WUE_WS_. This suggests that water deficit did not alter the relationship between DM accumulation and water use but reduced the proportion of the DM partitioned to sugar in the root. It has previously been identified that under drought phloem loading can be reduced, lowering the amount of sugar in the root but not in the plant overall (Mäck and Hoffmann, 2006). Secondly WUE_i_ was reduced compared to the fully irrigated in the Ltd treatment of the first measurement leaf in 2019 whilst it was generally higher in all other observations. Across the same time period as the reduced WUE_i_ the Ltd treatment also showed a greater C_i_/C_a_ ratio, whilst A_max_ and g_s_ were close to zero. An increase in C_i_ has been observed in extreme cases of water deficit previously and is driven by an increase in non-stomatal limitations to photosynthesis (Flexas and Medrano, 2002).

The measured gas exchange parameters recovered after drought, but only partially, due to the underlying leaf age related decline. Leaves respond differently to heat stress depending on age, with younger leaves showing responses of a greater magnitude compared to older leaves (Marias et al., 2017). This occurs because shading of older leaves drives reallocation of Nitrogen from Rubisco to light harvesting proteins reducing the maximum photosynthetic rate (Sims and Pearcy, 1991). However, when looking at the overlap of measurement leaf 1 and 2 from 105-133 DAS in 2018 it is evident that measurement leaf 1 reflected the trend of the response in measurement leaf 2 but not the magnitude. This means that, despite the overall decline in leaf activity with age, they still provided a reliable measure of the onset of drought and the subsequent recovery.

### 4.2 Does sugar beet acclimate to water deficit by increasing WUE?

Both sugar beet varieties responded similarly to the first and second drought, suggesting the crop did not acclimate to avoid or better cope with water deficit. This has also been observed in glasshouse studies where sugar beet was exposed to three temporary water deficits but no acclimation was evident in photosynthetic or biochemical responses (Leufen et al., 2016; Ebmeyer and Hoffmann, 2022). This recovery is mediated by a range of physiological and biochemical mechanisms. How these interact to enable plant survival and recovery under drought stress is not clear and requires further study (Chaves et al., 2009). The maritime ancestry of sugar beet (Ribeiro et al., 2016) may be a driver of this with the plants showing drought tolerance (Dunham, 1993) and the photosynthetic apparatus being able to withstand severe water deficit and recover rapidly (Monti et al., 2006). This resilience is partly driven by the accumulation of K, Na and AN, as observed in 2019, to maintain osmotic potential for longer to ensure cell function. This means there is no need to avoid a decline in leaf RWC, as the plant can continue to photosynthesize until the most severe levels of drought. Even then no long-term damage occurs to the PSII, as shown by the recovery of the F_v_’/F_m_’, ΦPSII and q_p_ values which returned to levels similar to the fully irrigated once re watered. However, the recovery of maximum PSII efficiency in the light, and A_max_, was not immediate and may have contributed to the reduced DM accumulation in the droughted treatments as evidenced in sugar beet by Bloch et al. (2006). It has been suggested that measuring photosynthetic induction coupled with A_max_ provides better data on photosynthesis and would have been a valuable approach in this study. This is because induction can provide a performance in the field under fluctuating light regimes whereas A_max_ is not always as sensitive to drought stress, especially during recovery (Sakoda et al., 2022).

The lack of acclimation from the first drought was also reflected in the decline in WUE_i_ and increase in C_i_/C_a_ as the sugar beet opened stomata and began to reach similar levels of g_s_ and A_max_ to the fully irrigated treatment. The rapid recovery of leaf RWC, which recovered faster than PSII and leaf gas exchange, helps drive this recovery by ensuring the leaf has optimal conditions for photosynthesis (Lawlor, 2002), with the rapid recovery of RWC over daily cycles previously observed in sugar beet (Geiger et al., 1991). Not only was this recovery evident in these short-term measurements but also in the Δ^13^C results where the SD leaves showed no difference compared to the fully irrigated treatment and therefore no long-term change in WUE_i_.

Water deficit has been shown to alter the stomatal density of leaves which develop under drought (Xu and Zhou, 2008; Sun et al., 2014). There were no consistent changes in stomatal density highlighting further that sugar beet physiology changes little under water deficit. There is an exception to this which is the reduction in plant biomass which appears to have resulted in the crop having a reduced demand for water, likely due to a reduction in canopy size. Canopy size was not measured but water deficit has been shown to reduce leaf area in sugar beet (Fitters et al., 2017) which results in reduced radiation interception and DM accumulation (Brown et al., 1987). This was evidenced by the reduction in VWC in the fully irrigated treatment at 140 DAS which the SD did not encounter despite receiving the same amount of irrigation. This seems to have been beneficial under a slight water deficit but did not alter the response to the severe second drought in 2019. In the late second drought in 2018, the slight decline in A_max_ and g_s_ was not reflected in any other parameter and shows how late season drought can be hard to detect as the forces driving transpiration are reduced.

### 4.3 Are there differences in WUE between commercial sugar beet varieties with contrasting canopy architecture, and if so, is this related to stomatal morphology and leaf traits such as SLW and RWC?

The difference in Δ^13^C and associated traits between the two varieties supports the findings of Ober et al. (2004); Ober et al. (2005) that there is variation in traits associated with drought tolerance and water use in sugar beet, despite suggestions that sugar beet varieties lack diverse traits due to being derived from a single population (Davis, 2006). The varieties also differed in leaf physiology, but it is not clear if this can be attributed to the differences in canopy architecture and requires further study with more varieties.

The traits identified differing between the varieties suggest that the prostrate variety is more conservative in its use of water. The greater RWC is linked with more drought resistant phenotypes as a greater RWC can enable plants to function for longer under water deficit (Xu et al., 2000). Ober et al. (2005) did not find RWC to be linked to DTI but leaf succulence was. This suggests there is a relationship between the water content of leaves and drought tolerance, which this study has further shown to be linked to a greater WUE_i_. RWC did not reduce the rate of carbon uptake and assimilation as has been observed in wheat (Farquhar et al., 1980). This is supported by the observation that RWC is not the driver for reduced Rubisco activity and that reduced CO_2_ concentrations in the chloroplast due to reduced stomatal conductance have a greater regulatory effect (Flexas et al., 2006). This shows that stomata have an important role to play in plant carbon dynamics and therefore WUE.

The lower stomatal density in the prostrate variety may be associated with WUE_i_ but complex interactions between stomatal density and size and the speed of stomatal response means that the relationship between stomatal density and water use is debated. In potatoes a higher stomatal density, which developed under drought, led to a greater Δ^13^C and WUE_i_ (Sun et al., 2014) and it has been suggested the smaller stomata are faster to close and reduce transpiration and increase WUE_i_ (Drake et al., 2013; Barratt et al., 2021). However, in Arabidopsis a lower stomatal density was associated with reduced susceptibility to water deficit (Doheny-Adams et al., 2012), which could explain why the prostrate variety had a cooler canopy than the upright under extreme water deficit (Ltd treatment).

The difference in stomatal traits could be driven by the differences in canopy architecture. This is because the leaves of the upright variety are less shaded and intercept more light on the abaxial surface. This can result in a greater number of stomata on the abaxial surface of the upright variety to optimize CO_2_ uptake for photosynthesis (Harrison et al., 2020). This is supported by a study that used the same sugar beet varieties which showed that the upright sugar beet variety has a greater RUE than the prostrate (Tillier et al., 2023). Despite the difference in RUE, and as observed in this study, there was no significant difference in yield. The difference in stomatal number and potential link to photosynthetic performance was not supported by the spot gas exchange measurements as both varieties performed similarly. A difference could have been expected between the older measurement leaves as greater shading in the prostrate could result in a quicker decline in photosynthetic performance as N is reallocated from Rubisco as previously outlined. However, the observation that both varieties have similar photosynthetic performance has been made before (Tillier et al., 2023) and suggests that the prostrate variety performs as well as the upright despite intercepting less radiation to achieve an equal sugar yield.

Büssis et al. (2006) showed that increased stomatal aperture can compensate fewer stomata to enable similar levels of A and g_s_ but that under high light intensities g_s_ was reduced compared to the plants with a higher stomatal density. This may explain the higher canopy temperature in the prostrate variety in fully irrigated conditions as lower g_s_ rate results in reduced cooling by transpiration. This reduction in g_s_ was also observed in 2019, leading to a greater WUE_i_ which highlights how different stomatal densities may be suited to different environments with a lower density making a plant more prone to heat stress under higher temperatures and irradiance (Lu et al., 1998) although such conditions are only present for short periods in much of the temperate beet growing area. This also highlights the importance of understanding the genotype x environment interaction of different traits, as environmental conditions can significantly affect how traits drive sugar beet yield (Hoffmann et al., 2009). In addition to this other factors that drive photosynthetic performance and linked to stomatal traits must also be considered such as stomatal size and leaf mesophyll conductance (Gago et al., 2014; Bertolino et al., 2019).

The varieties had a similar SLW despite observing that a higher SLW was associated with a lower Δ^13^C and greater WUE_DM_, and this relationship did not vary depending on water deficit as had been previously observed (Rajabi et al., 2008). In this study SLW was correlated with Δ^13^C but this was driven by difference in SLW due to water deficit, with plants exposed to a greater water deficit having a lower Δ^13^C and WUE_DM_. This suggests that the strong relationship between SLW, Δ^13^C and WUE_DM_ observed by may not always be evident on a varietal basis and other factors may be more strongly correlated with WUE_DM_. Additionally, the observation of lower levels of K and Na in the prostrate variety suggest water regulation in the root was different to the upright which could be linked to the drought tolerant traits observed.

Overall, the prostrate variety was more efficient in its use of water and had a reduced stomatal density and greater RWC which are traits associated with increased WUE and drought tolerance. It is not known whether these traits are present in all sugar beet genotypes with prostate canopies, as only one was examined in this study, and this is an area for further research.

Leaf chlorophyll content was greater in the upright variety except in the Ltd treatment, where the prostrate variety had a similar chlorophyll content and a greater leaf water content suggesting that under severe and prolonged water deficit the leaf morphology had changed, which was not evident in the upright variety.

The increase in chlorophyll content was opposite to that typically observed under drought for many species such as maize (Din et al., 2011) and rice (Pirdashti et al., 2009) and there was no change in the ratio of chlorophyll *a* and *b* which is also sometimes observed (Saeidi and Zabihi-e-Mahmoodabad, 2009). However, an increase in chlorophyll content has previously been reported in sugar beet exposed to drought (Hussein et al., 2008) and increased SPAD values (a proxy for chlorophyll content) were observed in sugar beet exposed to high level water deficits by Fitters et al. (2018), although these differences were no longer present after re-watering and were attributed to a dilution effect. No such dilution effect is evident in this study as leaf water content was measured at the same time as collection of leaf discs for chlorophyll extraction. Additionally, the prostrate variety had a more stable chlorophyll content with an increase only evident in the Ltd treatment, whilst the upright variety was more variable, but this did not seem to affect the photosynthetic performance of the two varieties. In barley, varieties with a more stable chlorophyll content across varying moisture deficits were found to have greater drought tolerance (Li et al., 2006) but in wheat greater chlorophyll content was associated with greater drought tolerance (Talebi, 2011). The relationship between leaf chlorophyll content and WUE is therefore unclear in sugar beet and is an area for future research.

## 5 Conclusion

In conclusion, we have shown that water deficits tend to increase WUE_i_ and WUE_DM_ but reduce total yield. The recovery of sugar beet after drought has shown the crop’s resilience and ability to recover from even the most severe drought. It is evident that the crop does not acclimate after these events to increase WUE or avoid future water deficits, except for reducing its canopy size. The lower Δ^13^C of the prostrate variety and the difference in stomatal density and RWC compared to the upright suggests traits associated with greater WUE_i_ may be linked to canopy architecture. This should be confirmed by further research utilizing more varieties that contrast in canopy architecture.

## Data availability statement

The raw data supporting the conclusions of this article will be made available by the authors, without undue reservation.

## Author contributions

GB, EM and DS designed the experiment. GB carried out the practical work, statistical analysis and wrote the manuscript with contributions from EM and DS. EM supported the gas exchange measurements and analysis and DS the other crop physiology assessments. All authors met regularly to review the results and discuss management of the trial. All authors contributed to the article and approved the submitted version.
