# Supplementary material for: Water use efficiency responses to fluctuating soil water availability in contrasting commercial sugar beet varieties
Source: Front Plant Sci. 2023 Mar 9;14:1119321. doi: 10.3389/fpls.2023.1119321 (PMC10034331; doi:10.3389/fpls.2023.1119321)
Supplement: Supplementary file 1 [file DataSheet_1.pdf]

Supplementary Material

Water use efficiency responses to fluctuating soil water availability in contrasting commercial sugar beet varieties

Georgina E Barratt, Erik H Murchie, Debbie L Sparkes

Correspondence: Georgina Barratt: georgina.barratt@bbro.co.uk

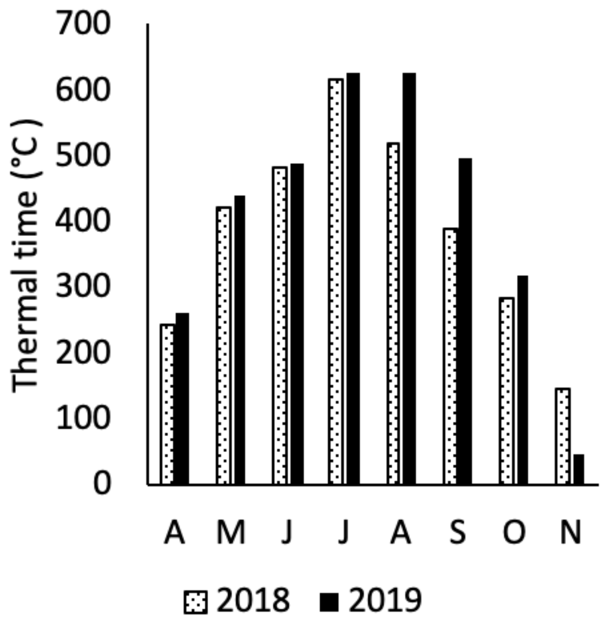

Fig S1. *Cumulative thermal time for each month from April to November in 2018 and 2019. The thermal time in November is not comparable as harvest was later in 2018.*

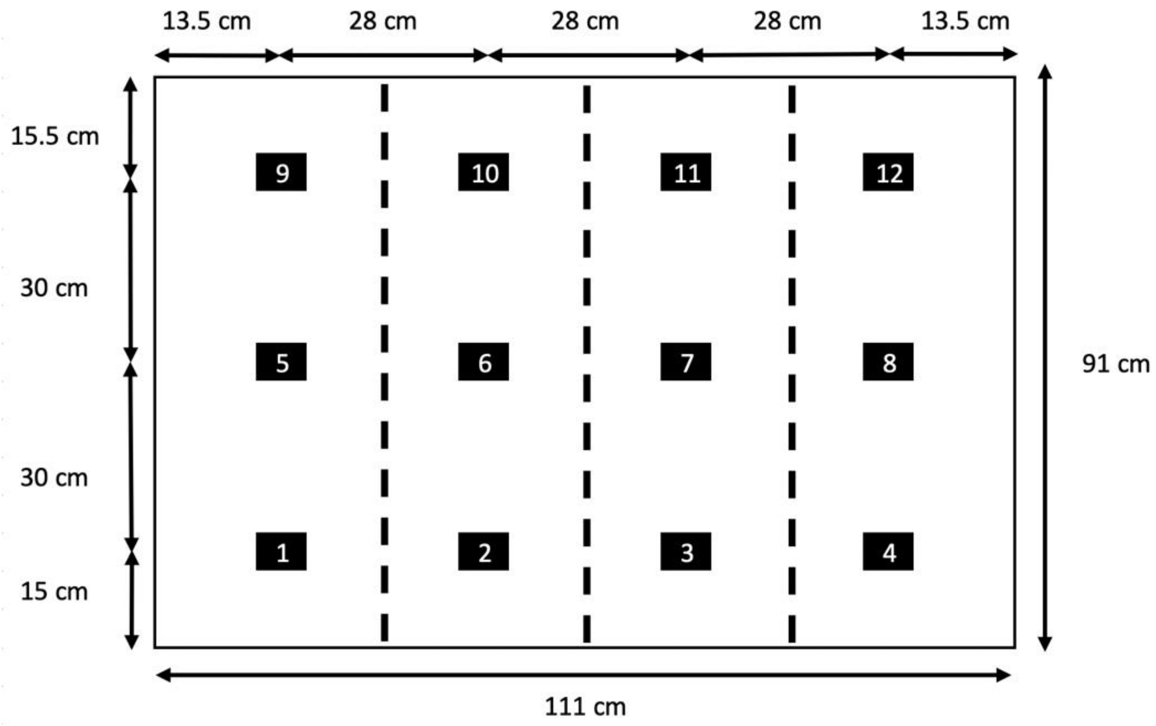

Fig S2. *The seed spacing for the boxes used to grow sugar beet to examine WUE, each numbered box represents a sowing location and the dashed lines represent the location of the drip irrigation pipes.*

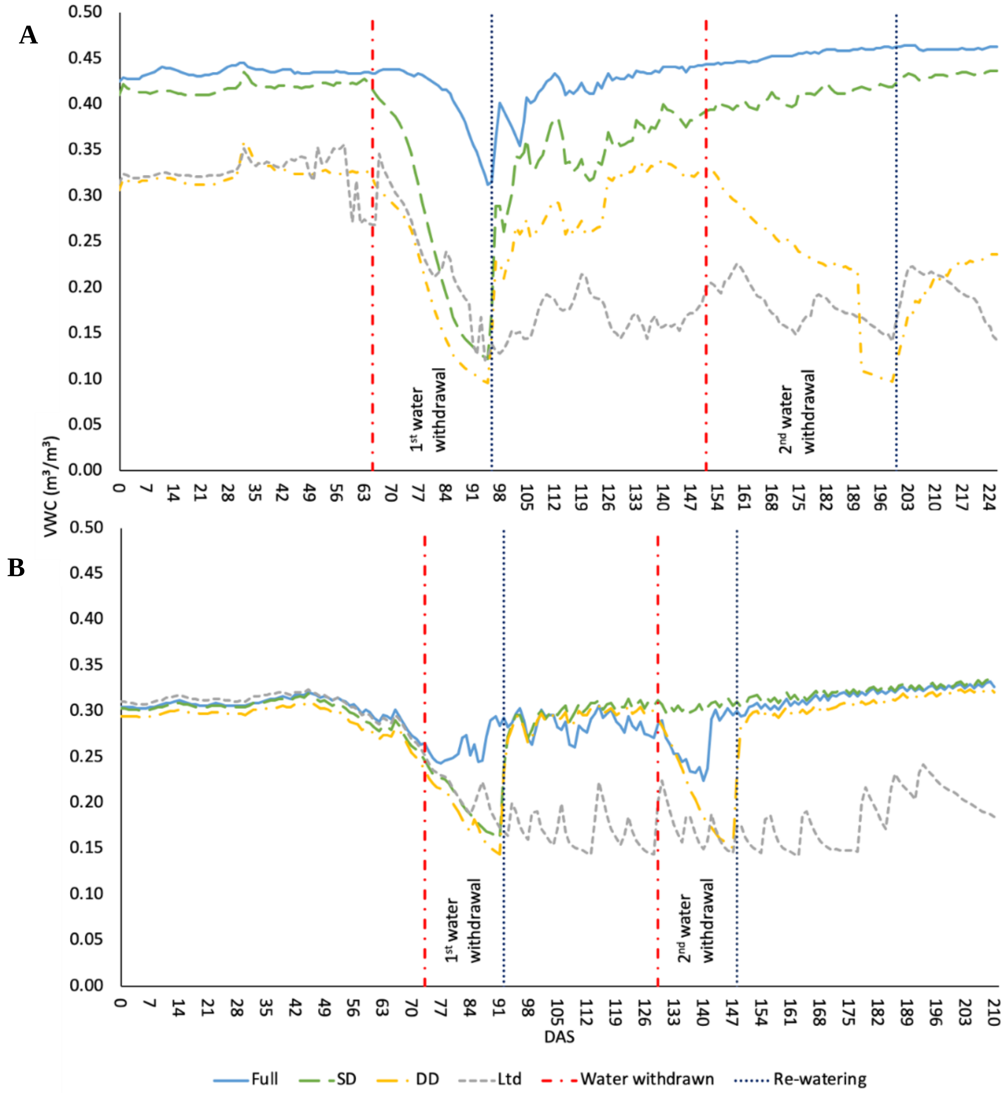

Fig S3. *The average volumetric water content (VWC) of soil in 610L boxes containg 12 sugar beet plants grown under 4 different irrgation regimes in 2018 (A) and 2019 (B). In 2018 water was withdrawn from 65 DAS to 96 DAS for the single drought (SD), whilst the double drought (DD) was exposed to a second water withdrawal period from 151 DAS to 200 DAS. In 2019 water was withdrawn from 73 DAS to 92 DAS for the single drought (SD), whilst the double drought (DD) was exposed to a second water withdrawal period from 129 DAS to 148 DAS. No water was withdrawn from the fully irrigated (F) or the water limited (Ltd) treatment boxes.*

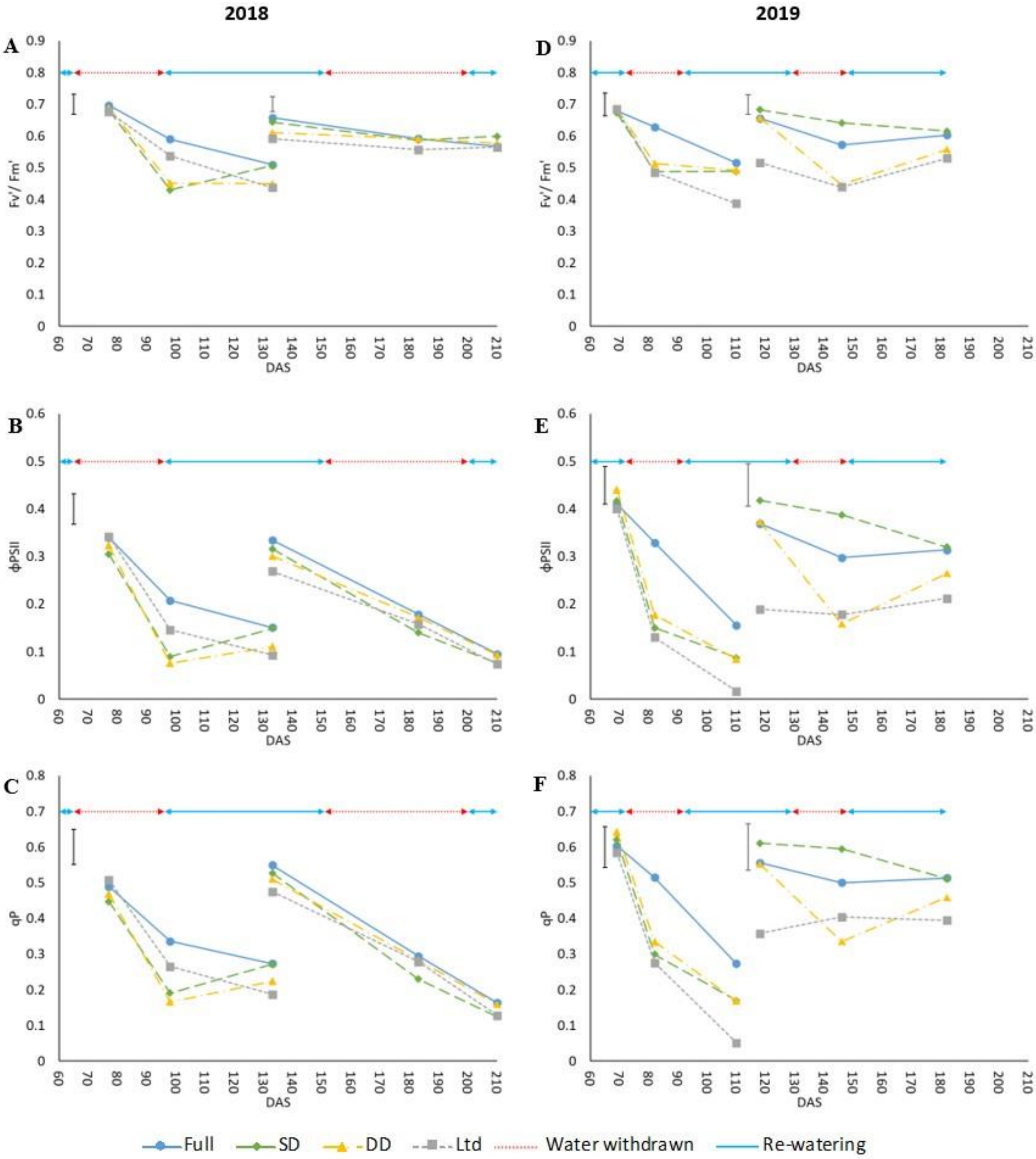

Fig S4. *The ϕPSII, Fv'/ Fm' and qP of sugar beet grown under four irrigation regimes, measured using an infra-red gas analyser (Li6800, LiCor, Nebraska, US). Measurements were taken from 2 leaves Measurement leaf 1 covers the first drought and measurement leaf 2 the second drought in 2018 and 2019. (A) leaf 1 (LSD=0.063 DF=190 P<0.001), leaf 2 (LSD=0.047 DF=191 P=0.017). (B) leaf 1 (LSD=0.064 DF=190 P<0.001), leaf 2 (P=0.144). (C) leaf 1 (LSD=0.098 DF=190 P=0.002), leaf 2 (P=0.254). (D) leaf 1 (LSD=0.071 DF=215 P<0.001), leaf 2 (LSD=0.061 DF=253 P<0.001). (E) leaf 1 (LSD=0.080 DF=207 P<0.001), leaf (LSD=0.089 DF=253 P<0.001). (F) leaf 1 (LSD=0.115*

DF=207 P<0.001), leaf 2 (LSD=0.130 DF=253 P=0.011). Error bars show time x irrigation LSD for all data points. ANOVA tables containing all the data points are available for ϕPSII and Fv'/ Fm' in supplementary Table S5 (2018) and Table S6 (2019). Irrigation regimes were a fully irrigated (Full), a continually water limited kept at approx. 50% field capacity (Ltd), a single drought (SD) (2018 65-96 DAS and 2019 73 -92 DAS) and a double drought (DD) which was exposed to the single drought treatment plus an additional drought (2018 151-200 DAS and 2019 118-182 DAS).

Table S5. *The A_{max}, g_s, WUE_i, Ci/Ca, Fv'/Fm', ϕPSII and qP of sugar beet grown under four irrigation regimes in 2018, measured using an infra-red gas analyser (Li6800, LiCor, Nebraska, US). Measurement leaf 1 covers the first drought and measurement leaf 2 the second drought. Irrigation regimes were a fully irrigated (Full), a continually water limited kept at approx. 50% field capacity (Ltd), a single drought (SD) (65-96) and a double drought (DD) which was exposed to the single drought treatment plus an additional drought (151-200 DAS).*

2018			Measurement Leaf 1						Measurement Leaf 2					
Parameter	Units	Treatment	77	92	98	105	113	133	105	113	133	183	196	210
A_{max}	µmol m^{-2} s^{-1}	Full	24.46	19.21	14.53	16.23	13.33	9.10	21.55	22.89	18.44	10.52	10.68	6.68
		SD	20.64	2.26	3.90	5.04	7.42	8.20	15.65	20.06	17.69	10.04	9.00	5.61
		DD	22.46	2.51	3.17	4.73	3.80	5.25	14.85	18.07	16.46	10.65	7.26	6.96
		Ltd	22.70	10.48	7.77	3.26	5.75	4.38	4.12	15.03	14.11	8.50	6.55	5.39
		Irrigation*Time	*DF*	*SS*	*MS*	*F*	*P*		*DF*	*SS*	*MS*	*F*	*P*	
			15	1147.02	76.47	5.68	<.001		15	788.35	52.56	3.48	0.002	
g_s	mol m^{-2} s^{-1}	Full	0.862	0.641	0.371	0.505	0.353	0.164	0.777	0.709	0.394	0.151	0.162	0.108
		SD	0.488	0.021	0.051	0.050	0.085	0.122	0.246	0.356	0.301	0.154	0.125	0.093
		DD	0.622	0.022	0.029	0.049	0.038	0.073	0.250	0.289	0.263	0.165	0.103	0.108
		Ltd	0.644	0.229	0.106	0.042	0.070	0.059	0.037	0.300	0.222	0.107	0.063	0.077
		Irrigation*Time	*DF*	*SS*	*MS*	*F*	*P*		*DF*	*SS*	*MS*	*F*	*P*	
			15	1.099	0.073	3.11	0.005		15	1.895	0.126	7.81	<.001	
WUE_i	µmol $C0_2$ mol^{-1} H_20	Full	32.1	40.2	57.2	56.6	55.9	70.9	42.1	36.6	58.3	74.0	70.4	64.5
		SD	54.9	102.6	75.7	116.7	111.8	78.5	90.8	69.6	69.8	75.3	79.2	61.9
		DD	46.2	104.2	109.6	111.3	116.5	80.9	83.0	73.9	74.6	77.7	74.1	70.3
		Ltd	47.3	86.9	96.1	102.9	128.3	82.0	123.2	82.6	73.7	86.9	107.2	75.8
		Irrigation*Time	*DF*	*SS*	*MS*	*F*	*P*		*DF*	*SS*	*MS*	*F*	*P*	
			15	22663.3	1510.9	2.76	0.004		15	18548.2	1236.5	3.25	0.004	
Ci/Ca		Full	0.820	0.794	0.730	0.728	0.736	0.685	0.781	0.803	0.729	0.667	0.685	0.716
		SD	0.727	0.563	0.668	0.499	0.516	0.655	0.584	0.667	0.684	0.663	0.653	0.727
		DD	0.762	0.557	0.534	0.521	0.505	0.648	0.618	0.651	0.666	0.653	0.676	0.692
		Ltd	0.757	0.615	0.580	0.557	0.454	0.645	0.474	0.624	0.671	0.618	0.543	0.672
		Irrigation*Time	*DF*	*SS*	*MS*	*F*	*P*		*DF*	*SS*	*MS*	*F*	*P*	
			15	0.333	0.022	2.63	0.006		15	0.284	0.019	3.24	0.004	
Fv'/Fm'		Full	0.697	0.657	0.591	0.585	0.583	0.510	0.639	0.658	0.580	0.592	0.639	0.567
		SD	0.681	0.454	0.430	0.428	0.514	0.507	0.564	0.644	0.585	0.587	0.641	0.600
		DD	0.688	0.497	0.452	0.434	0.461	0.450	0.566	0.612	0.573	0.592	0.617	0.578
		Ltd	0.677	0.590	0.537	0.421	0.514	0.438	0.470	0.592	0.545	0.557	0.601	0.565
		Irrigation*Time	*DF*	*SS*	*MS*	*F*	*P*		*DF*	*SS*	*MS*	*F*	*P*	
			15	0.232	0.015	5.57	<.001		15	0.074	0.005	2.37	0.017	
ϕPSII		Full	0.340	0.270	0.208	0.250	0.191	0.151	0.639	0.658	0.580	0.592	0.639	0.567
		SD	0.305	0.076	0.089	0.125	0.134	0.148	0.564	0.644	0.585	0.587	0.641	0.600
		DD	0.323	0.112	0.076	0.122	0.077	0.111	0.566	0.612	0.573	0.592	0.617	0.578
		Ltd	0.342	0.176	0.146	0.090	0.113	0.093	0.470	0.592	0.545	0.557	0.601	0.565
		Irrigation*Time	*DF*	*SS*	*MS*	*F*	*P*		*n.s*					
			15	0.175	0.012	4.34	<.001							
qP		Full	0.489	0.409	0.336	0.414	0.314	0.273	0.486	0.487	0.549	0.294	0.204	0.164
		SD	0.448	0.163	0.191	0.281	0.251	0.271	0.518	0.490	0.527	0.230	0.171	0.124
		DD	0.469	0.229	0.166	0.268	0.164	0.224	0.459	0.486	0.510	0.283	0.178	0.159
		Ltd	0.508	0.285	0.265	0.201	0.215	0.188	0.355	0.441	0.475	0.278	0.174	0.128
		Irrigation*Time	*DF*	*SS*	*MS*	*F*	*P*		*n.s*					
			15	0.324	0.022	3.41	0.002							

Table S6. *The A_{max}, g_s, WUE_i, Ci/Ca, Fv'/Fm', ϕPSII and qP of sugar beet grown under four irrigation regimes in 2019, measured using an infra-red gas analyser (Li6800, LiCor, Nebraska, US). Measurement leaf 1 covers the first drought and measurement leaf 2 the second drought. Irrigation regimes were a fully irrigated (Full), a continually water limited kept at approx. 50% field capacity (Ltd), a single drought (SD) (73 -92 DAS) and a double drought (DD) which was exposed to the single drought treatment plus an additional drought (118-182 DAS).*

**2019**			Measurement Leaf 1							Measurement Leaf 2							
Parameter	Units	Treatment	69 DAS	76 DAS	82 DAS	90 DAS	97 DAS	104 DAS	110 DAS	118 DAS	140 DAS	146 DAS	153 DAS	162 DAS	169 DAS	174 DAS	182 DAS
A_{max}	$\mu mol\ m^{-2}\ s^{-1}$	Full	28.43	24.17	14.67	19.28	15.41	10.06	7.27	21.93	13.77	14.48	18.34	16.34	17.05	13.22	14.98
		SD	28.59	20.24	2.03	0.93	3.44	3.35	3.86	23.95	22.61	20.29	19.70	18.04	16.06	15.98	15.86
		DD	30.07	23.54	3.10	1.35	3.31	4.60	3.99	22.34	7.15	4.10	9.27	9.07	12.27	12.53	12.15
		Ltd	28.34	20.33	1.97	2.29	1.11	0.92	0.83	6.44	3.18	3.81	4.46	3.23	7.77	8.85	7.90
	Irrigation*Time		*DF*	*SS*	*MS*	*F*	*P*			*DF*	*SS*	*MS*	*F*	*P*			
			18	1610.06	89.45	7.39	<.001			21	1602.14	76.29	6.22	<.001			
g_s	$mol\ m^{-2}\ s^{-1}$	Full	0.809	0.548	0.186	0.370	0.306	0.181	0.167	0.608	0.260	0.252	0.373	0.265	0.301	0.214	0.224
		SD	0.760	0.379	0.016	0.014	0.065	0.040	0.052	0.646	0.534	0.429	0.395	0.326	0.263	0.264	0.261
		DD	0.842	0.476	0.025	0.017	0.035	0.060	0.064	0.547	0.117	0.053	0.169	0.120	0.189	0.204	0.171
		Ltd	0.787	0.420	0.016	0.036	0.027	0.021	0.011	0.062	0.025	0.031	0.035	0.030	0.076	0.090	0.080
	Irrigation*Time		*n.s*							*DF*	*SS*	*MS*	*F*	*P*			
										21	1.349	0.064	5.08	<.001			
WUE_i	$\mu mol\ CO_2\ mol^{-1}\ H_2O$	Full	38.6	52.6	99.1	68.7	65.5	87.1	80.3	46	71.3	75.3	59.4	70.8	65.8	74.3	72.2
		SD	42.1	65.3	120.3	66.8	84.9	95	80.8	46.2	49.6	57.8	61.2	64.4	69.9	71.2	68.3
		DD	38.2	59.4	126.7	74.2	96.9	90.6	93.4	51.7	81.8	66	83.3	102.5	77.6	77.7	86.3
		Ltd	37.3	66.2	98.4	93.3	44.9	38.4	61.1	114.8	118.3	124	128	116.4	114.5	105.1	118.5
	Irrigation*Time		*DF*	*SS*	*MS*	*F*	*P*			*DF*	*SS*	*MS*	*F*	*P*			
			18	29503.9	1639.1	2.94	0.003			21	13822.0	658.2	2.14	0.022			
Ci/Ca		Full	0.787	0.735	0.558	0.676	0.692	0.614	0.649	0.766	0.672	0.656	0.716	0.671	0.690	0.663	0.668
		SD	0.771	0.688	0.496	0.711	0.633	0.592	0.651	0.761	0.747	0.719	0.706	0.694	0.675	0.670	0.683
		DD	0.786	0.707	0.469	0.680	0.583	0.608	0.601	0.742	0.639	0.707	0.634	0.555	0.650	0.651	0.616
		Ltd	0.792	0.685	0.583	0.603	0.792	0.820	0.735	0.508	0.499	0.478	0.461	0.510	0.508	0.544	0.493
	Irrigation*Time		*DF*	*SS*	*MS*	*F*	*P*			*DF*	*SS*	*MS*	*F*	*P*			
			18	0.465	0.026	3	0.003			21	0.198	0.009	2.09	0.027			
Fv'/Fm'		Full	0.680	0.657	0.628	0.636	0.603	0.515	0.515	0.657	0.584	0.572	0.612	0.614	0.609	0.574	0.603
		SD	0.673	0.626	0.487	0.472	0.420	0.405	0.489	0.683	0.667	0.641	0.638	0.619	0.612	0.600	0.615
		DD	0.686	0.649	0.513	0.476	0.401	0.409	0.493	0.658	0.466	0.446	0.475	0.513	0.570	0.569	0.558
		Ltd	0.685	0.620	0.486	0.465	0.368	0.346	0.387	0.516	0.420	0.439	0.476	0.449	0.516	0.531	0.530
	Irrigation*Time		*DF*	*SS*	*MS*	*F*	*P*			*DF*	*SS*	*MS*	*F*	*P*			
			18	0.261	0.014	4.7	<.001			21	0.241	0.011	5.63	<.001			
ϕPSII		Full	0.411	0.392	0.329	0.337	0.286	0.202	0.156	0.369	0.299	0.298	0.346	0.352	0.343	0.271	0.314
		SD	0.417	0.343	0.150	0.034	0.111	0.107	0.087	0.418	0.414	0.388	0.367	0.351	0.316	0.308	0.320
		DD	0.441	0.393	0.178	0.054	0.107	0.105	0.085	0.374	0.204	0.158	0.179	0.229	0.261	0.233	0.264
		Ltd	0.401	0.352	0.130	0.067	0.056	0.028	0.017	0.189	0.146	0.178	0.164	0.168	0.213	0.228	0.212
	Irrigation*Time		*DF*	*SS*	*MS*	*F*	*P*			*DF*	*SS*	*MS*	*F*	*P*			
			18	0.416	0.023	6.28	<.001			21	0.309	0.015	4.2	<.001			
qP		Full	0.604	0.593	0.515	0.516	0.455	0.362	0.273	0.556	0.488	0.500	0.561	0.568	0.561	0.462	0.514
		SD	0.620	0.540	0.299	0.073	0.261	0.251	0.170	0.611	0.606	0.595	0.569	0.561	0.505	0.501	0.511
		DD	0.643	0.601	0.335	0.111	0.259	0.233	0.169	0.553	0.400	0.336	0.336	0.426	0.442	0.400	0.459
		Ltd	0.585	0.552	0.275	0.135	0.128	0.072	0.052	0.358	0.331	0.404	0.343	0.371	0.406	0.427	0.395
	Irrigation*Time		*DF*	*SS*	*MS*	*F*	*P*			*DF*	*SS*	*MS*	*F*	*P*			
			18	0.902	0.050	5.9	<.001			21	0.433	0.021	2.61	0.011			

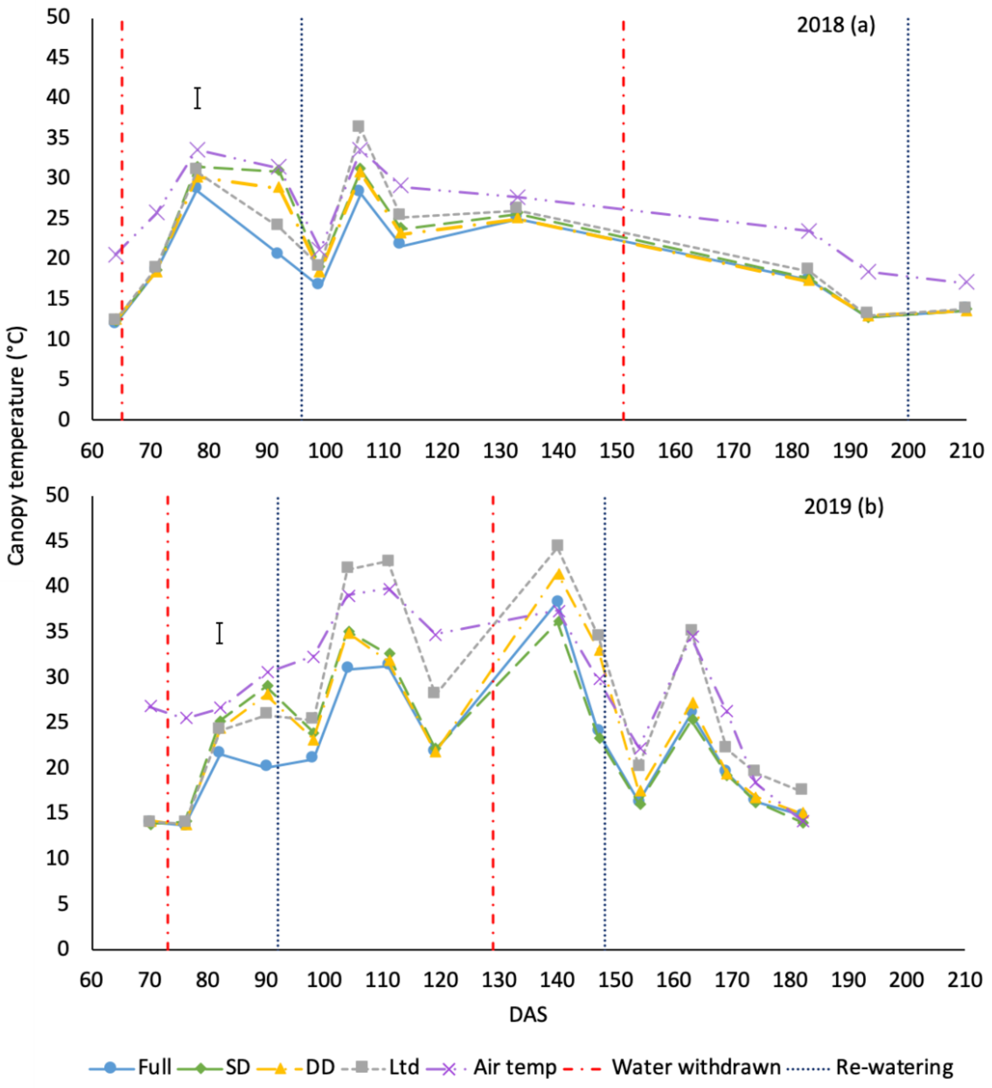

Fig S7. *The canopy temperature of sugar beet grown under four different irrigation regimes in 2018 (a) (LSD=2.56 P<0.001) and 2019 (b) (LSD=2.23 DF=42 P<0.001) and the air temperature at the time of measurement. Error bars show irrigation*time interaction. Irrigation regimes were a fully irrigated (Full), a continually water limited kept at approx. 50% field capacity (Ltd), a single drought (SD) (2018 65-96 DAS and 2019 73 -92 DAS) and a double drought (DD) which was exposed to the single drought treatment plus an additional drought (2018 151-200 DAS and 2019 118-182 DAS).*

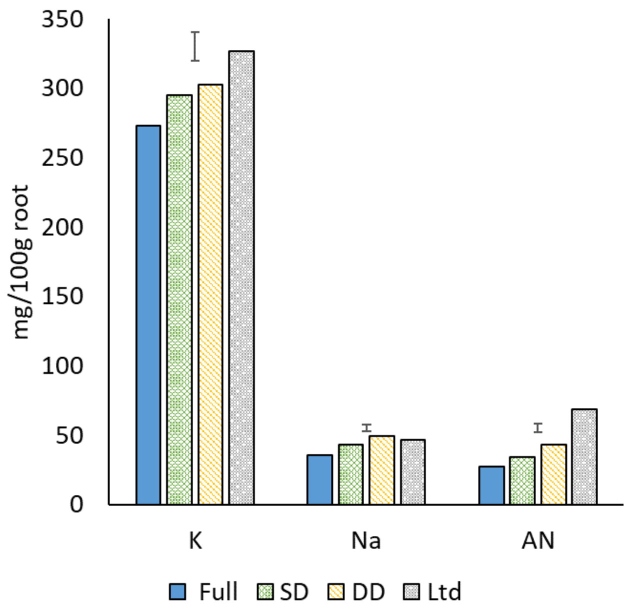

Fig S8. *The impurities (K, Na and AN) of sugar beet grown under four different irrigation regimes in 2019. K (P=0.002 DF=31 LSD=20.6), Na (P<0.001 DF=31 LSD=4.8) and AN (P=0.002 DF=31 LSD=6.2). Error bars show irrigation LSD.*

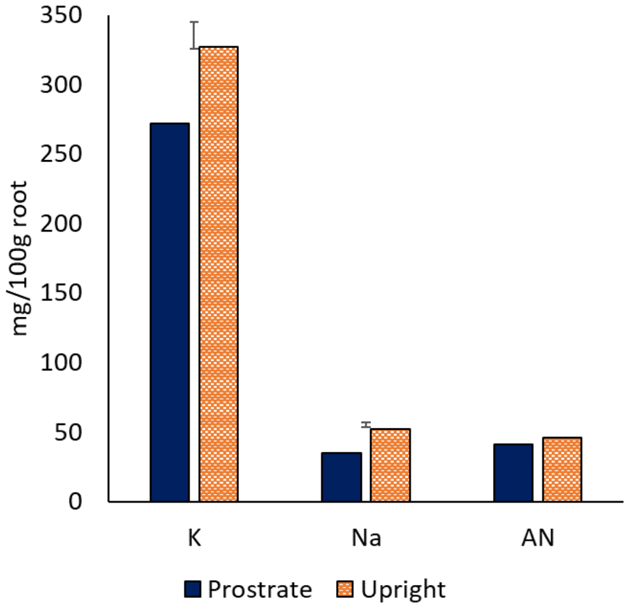

Fig S9. *The average impurities (K, Na and AN) of two sugar beet varieties grown under four different irrigation regimes in 2019. K (P<0.001 DF=31 LSD=9.8), Na (P<0.001 DF=31 LSD=1.7) and AN n.s. Error bars show variety LSD.*
